# Reliable on-treatment prognostication and target identification with a customized assay for circulating tumor DNA in patients with newly diagnosed pancreatic cancer

**DOI:** 10.1038/s41598-025-22369-5

**Published:** 2025-10-03

**Authors:** Alexandra Petersson, Maja Svensson, Sofie Olsson Hau, Rebecka Bergström, Johan Lindberg, Markus Mayrhofer, Subhayan Chattopadhyay, Jakob Eberhard, Markus Heidenblad, Karin Leandersson, David Gisselsson, Karin Jirström

**Affiliations:** 1 Division of Oncology and Therapeutic Pathology, Department of Clinical Sciences Lund, Lund University, Lund, 221 85 Sweden; 2 Department of Haematology, Oncology and Radiation Physics, Skåne University Hospital, Lund, Sweden; 3 Department of Medical Epidemiology and Biostatistics, Karolinska Institute, Stockholm, Sweden; 4 National Bioinformatics Infrastructure Sweden and Science for Life Laboratory, Department of Cell and Molecular Biology, Uppsala University, Uppsala, Sweden; 5 Division of Clinical Genetics, Department of Laboratory Medicine, Lund University, Lund, Sweden; 6 Center for Translational Genomics, Lund University, Lund, Sweden; 7 Clinical Genomics Lund, SciLifeLab, Lund, Sweden; 8 Cancer Immunology, Department of Translational Medicine, Lund University, Malmö, Sweden; 9 Department of Clinical Genetics, Pathology and Molecular Diagnostics, Skåne University Hospital, Lund, Sweden

**Keywords:** Pancreatic cancer, Chemotherapy, CtDNA, On-treatment monitoring, Prognosis, Cancer genomics, Gastrointestinal cancer, Pancreatic cancer

## Abstract

The vast majority of patients with pancreatic cancer present with unresectable disease and precision medicine is lagging behind. Circulating tumor DNA (ctDNA) has emerged as a promising tool, both as a proxy for tumor burden and for capturing tumor heterogeneity, but optimal gene panels and prognostic cutoffs remain to be determined. Herein, we applied ultra-deep ctDNA sequencing using a customized panel targeting 23 genes and six frequently altered chromosomes on plasma samples obtained before, during and after chemotherapy from 60 patients enrolled in a prospective clinical study. At baseline, positive versus negative ctDNA was not prognostic, neither in the adjuvant nor in the palliative setting, but in palliative patients, an independent prognostic cutoff could be calculated from the absolute number of mutated DNA molecules. Median overall survival was 3.7 months in the ctDNA^high^ compared to 11.9 months in the ctDNA^low^ group (*p* < 0.0001), and the cutoff remained prognostic at one and three months. Moreover, relevant genetic alterations were highly concordant in ctDNA and paired tumor tissue. These findings demonstrate the potential clinical utility of a customized and focused gene panel for prognostication and target identification over time in patients with newly diagnosed pancreatic cancer, in particular in the palliative setting.

ClinicalTrials.gov number: NCT03724994.

**Supplementary Information:**

The online version contains supplementary material available at 10.1038/s41598-025-22369-5.

## Introduction

The advancements in personalized medicine in the past decade have had a considerable positive effect on the outcome of patients with cancer, in particular treatments boosting the patient’s own immune response or targeting products of specific gene mutations. These therapies have, however, not yet demonstrated sufficient efficacy to earn a place in the treatment of pancreatic ductal adenocarcinoma (PDAC). Thus, systemic treatment options for patients with PDAC still rely on different types of chemotherapy, with only a modest effect on overall survival (OS)^1^. However, recent research suggests that up to 25% of PDAC cases harbor potentially actionable targets and that patients receiving molecularly matched therapy have an improved OS^2,3^. Despite these promising findings, molecular testing of PDAC remains a clinical rarity. Limited access to tumor tissue, especially in patients with advanced disease, and the underestimation of spatial tumor heterogeneity, might partly account for the difficulty in taking further steps towards personalized treatment.

The restricted tissue availability has spurred the exploration of liquid biopsies and their clinical utility in PDAC, in particular as recent research has proposed their superiority to tumor tissue analyses in capturing tumor heterogeneity and therapy resistance^4^. Circulating tumor DNA (ctDNA), i.e. the cancer-derived plasma proportion of cell free DNA (cfDNA), has emerged as a particularly promising clinical tool^5–8^. Sensitive detection methods, such as digital droplet PCR (ddPCR) and next generation sequencing (NGS), have enabled analyses of ctDNA, often with a primary focus on the *KRAS* gene^7–10^, as this is mutated in about 90% of PDAC^11,12^. Longitudinal examination of *KRAS* mutated ctDNA, as a proxy for tumor burden and chemotherapy resistance, has been compared to the clinically established serum marker carbohydrate antigen 19 − 9 (CA19-9) and imaging data, with promising results^10,13–15^. In addition, previous studies on tumor dynamics of PDAC encourage the use of ctDNA for precision medicine^16–18^. Several studies have also demonstrated a negative association between ctDNA levels in plasma before treatment initiation and prognosis^6,7,10,15^, although without any clear consensus regarding a clinically meaningful cutoff. Thus, the clinical value of ctDNA quantity and monitoring merits further study, particularly for patients with palliative disease where ctDNA has been detected in the majority of patients^7,10,15^.

This study aimed to develop a targeted sequencing approach with a gene panel that is customized for use in clinical routine, incorporating genes with actionable targets. This panel was applied in a real-world setting with longitudinal plasma samples from 60 patients with PDAC or other pancreatobiliary-type periampullary adenocarcinoma enrolled in a prospective, observational clinical study^19^. The goal was to identify clinically relevant dynamics in the quantity and characteristics of ctDNA during systemic chemotherapy, and to assess the concordance with genomic tissue profiling.

## Methods

### Study cohort

The cohort in the present study includes all patients enrolled in the prospective, observational single-arm study CHAMP (clinicaltrials.gov; NCT03724994) up until the 31st of December 2020 (*n* = 60)^19,20^. The study invited patients with a confirmed diagnosis of pancreatic or other periampullary adenocarcinoma receiving neoadjuvant, adjuvant or first-line palliative chemotherapy treatment at the Department of Oncology, Skåne University Hospital. Clinical and radiological follow-up was performed according to standard clinical protocols. All biopsies and resected tumors were re-evaluated by a senior board-certified pathologist (KJ). Clinical data, including routine laboratory parameters, were collected from patient records, as previously described^20^. Follow-up time ended either at date of death or last follow-up on December 31st, 2024. In September 2021, a sub-study with research autopsies was initiated, to which patients are invited to participate when they are in a late palliative phase. All autopsies are performed by a senior pathologist (KJ). Only one patient in the present study (*n* = 60) has been autopsied.

### Ethics declaration

Ethical approval for the CHAMP study has been obtained by the Regional Ethical Review Board (dnr LU 2018/13) and the Swedish Ethical Review Authority (amendments 2021 − 00166 and 2021–06065). Before entry, the patients receive information about the study from their oncologist or research nurse and provide a written informed consent if they want to participate. The patients are informed about the autopsy study when they are in a late palliative phase and provide a separate written informed consent for this part of the study. Of note, no change in treatment regimen is performed due to study participation, and the study protocol follows the agreements of the Declaration of Helsinki.

### Blood sampling

Serial blood samples were obtained before the start of each chemotherapy cycle and after the end of treatment. At each time point, whole blood was collected in four EDTA tubes (up to 24 ml) for subsequent plasma and buffy coat isolation, following 30 min incubation at room temperature (RT). After each incubation, blood components were fractionated by centrifugation at 2 000 *g* for 10 min. Isolated buffy coat (150 µl) was added to a cell freezing solution (10% DMSO in fetal bovine serum, ratio 1:10) before storage in -80 °C. Plasma samples were centrifuged a second time at 14 000 *g* for 10 min, either before or after storage in -80 °C, to minimize genomic DNA contamination (Supplementary Table S1).

### Tumor tissue sampling and DNA extraction

Following standard deparaffinization, tissue derived DNA from resected specimens (15 patients, 90 samples), excision biopsies (2 patients, 5 samples), postmortem collected tumor tissue (1 patient, 10 samples) and diagnostic core needle biopsies of sufficient size (23 patients, 37 samples), was extracted using the Allprep DNA/RNA FFPE kit (Qiagen, Hilden, Germany) and QIAcube instrument (Qiagen) according to the manufacturer’s instructions, eluted in 25 µl buffer EB (Qiagen). DNA concentrations were determined using the Qubit fluorometer with the Trade mark^TM^ 1X dsDNA broad range assay kit (Invitrogen, Carlsbad, CA, US) and stored at -20 °C until further analyses (Supplementary Table S2).

### Extraction of genomic DNA for germline controls

Extraction of genomic normal DNA was performed from buffy coat samples for all patients (*n* = 60). The frozen buffy coat was thawed, diluted with PBS and centrifuged at 1200 *g* for 5 min followed by removal of the supernatant. The cell pellet was resuspended in 200 µl PBS before automated DNA extraction using the DNeasy blood and tissue kit (Qiagen) according to the manufacturer’s instructions (Spin-column protocol) with the QIAcube instrument. The extracted genomic DNA was eluted in 25 µl buffer EB, followed by quantification using the Qubit^™^ 1X dsDNA high sensitivity (HS) assay kit and storage at − 20 °C.

### Cell-free DNA isolation from plasma

For the majority (195/201) of the plasma samples, cfDNA was extracted in-house by utilization of the QIAVac 24 vacuum manifold (Qiagen) and the QIAamp Circulating Nucleic Acid kit (Qiagen), with 40 µl elution volume (EB buffer) according to standard protocols, without addition of carrier-RNA. cfDNA concentrations were determined using the Qubit^™^ 1X dsDNA HS assay kit, and to ensure minimal contamination of genomic DNA, the Cell-free DNA ScreenTape analysis (Agilent, Santa Clara, CA, US) was carried out with the Agilent 4200 TapesStation system (Agilent) at the Center for Translational Genomics, Lund University and Clinical Genomics Lund, SciLifelab.

### Genomic DNA library preparation and sequencing

For tumor tissue samples (*n* = 94) and buffy coat samples (*n* = 60), library preparation was performed on 100 ng genomic DNA using the KAPA HyperPlus kit (Roche Sequencing, Basel, Switzerland) according to the manufacturer’s instructions, at the Center for Translational Genomics, Lund University and Clinical Genomics Lund, SciLifelab. Fragmentation time was adjusted depending on the input sample type (tissue or buffy coat derived DNA) to achieve an average fragment length of sequencing libraries of 400 bp. xGen CS Adapters (Integrated DNA Technologies, IDT, Coralville, IA, US) in combination with xGen UDI Primer Pairs (IDT) were used for indexing. Generated libraries were enriched by hybridization capture using a customized pan-cancer biotinylated probe panel (GMCK panel, 2.4 Mb, details in Supplementary Table S3; TWIST Bioscience, San Francisco, CA, US), including coding regions of 197 genes, 132 gene hotspots and 2814 genome wide distributed single nucleotide polymorphisms (SNPs). Target enrichment was performed on 8-plex pooled libraries according to the manufacturer’s recommendations for probe panel size. Libraries were sequenced on the Novaseq 6000 platform (Illumina, San Diego, CA, US) with 2 × 150 bp paired end reads. The aim was a sequencing depth of 80 M paired-end reads per tissue sample and 25 M paired-end reads per buffy coat sample (see Supplementary Tables S2 and S4).

### Sequencing raw data analysis of tumor tissue and matched blood normal

Raw data analysis of tumor and matched normal blood samples were performed using the in-house Autoseq bioinformatic pipeline, integrating both commonly used and several in-house developed bioinformatic tools. FASTQ files were trimmed using Skewer^21^ to eliminate adapters, followed by extraction and annotation of UMIs back to the raw reads in an unmapped BAM format by using *FastqToBam* from fgbio (http://fulcrumgenomics.github.io/fgbio/). The unmapped BAM files were then converted back to FASTQ format, using the *SamToFastq* tool in Picard^22^, followed by alignment using BWA MEM^23^. UMI tags were then retrieved from the unmapped BAM to annotate the mapped BAM using the *MergeBamAlignment* in Picard and followed by realignment of reads around indels using GATK3’s *RealignerTargetCreator* and *IndelRealigner3*^24^. The final raw BAM file was further processed in two parallel pipelines for subsequent variant calling, one with deduplication by position using *MarkDuplicates* in Picard, and one where UMI based consensus reads were produced using *GroupReadsByUmi* followed by *CallDuplexConsensusReads* from fgbio. Realignment of the consensus reads was then performed once more, similarly to the raw read alignment, before refinement by using *FilterConsensusReads* and *ClipBam* from fgbio.

### Somatic variant calling in tumor tissue

Somatic variant calling of single nucleotide variants (SNVs) and insertions and deletions (InDels) in the tumor samples were performed using GATK Mutect2^25,26^, Strelka^27^, Vardict^28^ and VarScan2^29^ on the final consensus BAM file, followed by merging of the variants using SomaticSeq^30^ and annotation using the Ensembl Variant Effect Predictor (VEP), only including canonical transcripts^31^. Only variants called by two or more somatic callers, annotated with a high or moderate impact and a variant allele frequency (VAF) above 3% were used in downstream analyses. Manual curation of all variants were also performed using the Integrative Genomics Viewer (IGV, version 2.15.2)^32^ to ensure variant support in both sequencing directions and sufficient mapping quality. However, exceptions were made for variants detected in ctDNA or a different tumor sample from the same patient to avoid the introduction of false heterogeneity.

### Germline variant calling

For germline SNVs and InDels in normal buffy coat samples, the *HaplotypeCaller* from GATK^25,33^ and Strelka^27^ were utilized, followed by merging using *CombineVariants* from GATK and VEP annotation. Only variants called by *HaplotypeCaller*, with a high or moderate impact, a VAF above 25% and a gnomAD VAF^34^ of less than 3% were included in downstream analyses. In addition, only variants classified as either pathogenic or likely pathogenic, without conflicts and with a neoplastic association in ClinVar^35^, were reported.

### Copy number alterations

For calling copy number alterations (CNAs) in tumor tissue, we utilized information from both broad targeted NGS and the genome-wide SNP array Oncoscan Assay (Affymetrix, Santa Clara, CA, US). For the NGS data, CNVkit^36^, utilizing the position-deduplicated BAM files, was used. The Oncoscan CNV assay was utilized for a total of 128 tissue samples, at either the Array and Analysis Facility, Department of Medical Sciences, Uppsala University, or Eurofins Genomics Europe Genotyping A/S, Galten, Denmark, according to standard practice. The raw data analysis was performed as previously described^37^, using reference genome build hg19, and the final OSCHP-files were examined using the Nexus Copy Number 10.0 (BioDiscovery, El Segundo, CA, US) together with the Tumor Aberration Prediction Suite (TAPS)^38^, enabling visualization of the allelic composition and CNA heterogeneity. Calculations of the mutated sample fraction of each CNA was performed as previously described^37^.

### Customized ctDNA design – the CHAMP panel

A customized smaller sequencing panel, the CHAMP-panel, utilizing duplex UMIs for improved error correction, was developed for the present study to enable an ultra-sensitive and cost-effective on-treatment evaluation of ctDNA fluctuations and mutations. The CHAMP-panel was constructed as two separate probe panels, panel A (106 kb) and panel B (140 kb), with 1x tiling to provide > 99% coverage of the genomic regions, except for hotspot regions of the *KRAS* gene, which were designed with 2x tiling (TWIST Bioscience, panel design in Supplementary Tables S5-S7). In panel A, coding regions of 22 genes of particular interest were included, together with hotspot regions in *KRAS* (including codons G12, G13 and Q61). The genes were chosen based on variants detected in previous studies, including both recurrent variants detected in PDACs of different disease stages^39^ and ctDNA variants reported in samples from patients with locally advanced or metastatic disease^40^, together with genes known to recurrently harbor clinically actionable mutations. Panel B on the other hand was designed to enable detection of CNAs, such as deletions and amplifications, on frequently affected chromosomes, as several studies, including a previous study from our group, have reported high occurrence of chromosomal imbalances in PDAC^11,37,41^. Thus, panel B comprises a SNP backbone with probes for a total of 1130 SNPs, distributed on chromosome 3, 7, 8, 9, 17 and 18, together with 60 probes up and downstream of the *CDKN2A* gene to enable a more sensitive detection of the frequently seen homozygous deletion in this genomic region.

### Cell-free DNA library preparation and sequencing

For most (196/201) cfDNA samples, cfDNA was converted into sequencing libraries using the KAPA HyperPrep kit (Roche Sequencing) without prior fragmentation. The cfDNA input to the end repair/A-tailing reaction varied between 14,5 and 77,5 ng (median of 45 ng, see Supplementary Table S1). Ligation was performed overnight at 4 °C using xGen CS Adapters containing UMIs (2 × 3 bp, IDT) with an adapter:insert molar ratio of 200:1 as per the manufacturer’s recommendations. Indexing PCR was performed for 5 cycles with xGen UDI Primer Pairs (IDT). Generated libraries were enriched by normalization capture using a combination of the two custom biotinylated probe panels, A and B. Probe pool B was diluted 10-fold prior to pooling with probe pool A to optimize the coverage of the targeted coding regions. Target enrichment was performed on 8-plex pooled libraries according to the manufacturer’s recommendations for the probe panel size. Libraries were sequenced on the Novaseq 6000 platform (Illumina) with 150 bp paired-end reads, aiming at a sequencing depth of 40 M paired-end reads per sample. In addition, to enable distinction of blood variants associated with clonal hematopoiesis, generated libraries from the buffy coat derived genomic DNA were also enriched using the CHAMP probe panel and sequenced again in the same manner as the cfDNA samples.

### Ultra-deep sequencing raw data analysis

For ultra-deep sequencing raw data analysis of the cfDNA samples and germline controls, UMI tags were extracted from raw reads by converting FASTQ files to unmapped BAM files, with the read structure definition “3M2S + T 3M2S + T”, using fgbio’s *FastqToBam* (v. 2.1.0). These reads were then aligned to the GRCh37 reference genome (human_g1k_v37_decoy) using BWA MEM (v. 0.7.17)^23^. Sequences originating from the same molecule were grouped with fgbio’s *GroupReadsByUmi*, using the paired strategy, and consensus reads were generated using fgbio’s *CallDuplexConsensusReads*, excluding bases in the individual reads with a quality phred score below 30. The consensus reads were then again aligned to the same reference genome and filtered using fgbio’s *FilterConsensusReads* using the following cutoffs: min-reads = 2 1 1 (requiring every consensus read to be represented by at least one read from each strand), max-base-error-rate = 0.1 0.2, min-base-quality = 30 and require-single-strand-agreement = true. Finally, to avoid counting potential mutations in the overlap twice during variant calling the consensus reads were clipped using fgbio’s *ClipBam* to remove overlapping parts between read pairs.

In total, for the 196 cfDNA samples that were sequenced using the CHAMP-panel, the mean raw number of read pairs was 52.3 million (range 22.0-86.7) and the mean filtered UMI-collapsed target coverage was 1383 x (range 486–2331 x), with a median of 83% of target regions being covered by a minimum of 1000 UMI-collapsed reads (Supplementary Table S8). For deep sequencing of the normal leukocyte derived samples (*n* = 60), the mean raw number of read pairs was 57.4 million (range 39.7–93.1), mean filtered UMI-collapsed target coverage was 1586 x (range 1034–2416 x), and a median of 95% of target regions were covered by a minimum of 1000 UMI-collapsed reads (Supplementary Table S9).

### ctDNA variant calling

Variant calling of SNVs and InDels in cfDNA was performed over the panel design (panel A of the CHAMP probe panel), with 20 additional bps flanking each target region, using freebayes2 (v 1.3.7)^42^ and vardict-java (v 1.8.3)^28^, both with cutoffs of at least 3 supporting reads and a variant frequency above 0.1%. Variants were decomposed and normalized using bcftools *norm* (v 1.15)^43^ and variants from the two different callers were merged using bcftools *merge*. Subsequently, these were annotated using Ensembl VEP (v 104) prior to manual curation using IGV (version 2.15.2)^32^ to confirm appropriate direction evidence and adequate read alignment characteristics. Only variants supported by a minimum of five consensus reads, with no evidence of detection in the matched normal sample and annotated as canonical by VEP, were included in downstream analyses. Exceptions were made for variants detected in matched tumor tissue, in another plasma sample within a patient or for hotspot codons of *KRAS*, where VAFs below the limit of detection (LOD) were accepted to avoid introduction of type II errors. One mutated *TP53* allele was, however, detected in the normal sample of patient M25 (VAF 0.08%), but the variant was retained in downstream analyses as it was detected with a VAF of above 7% in the baseline (BL) plasma sample and in high frequencies in the matched tumor tissue.

### Copy number analysis of cfDNA

Calling of CNAs in cfDNA was performed using the bioinformatics tool Jumble^44^ for all targeted regions covered in both panel A and B. Thus, for this analysis, BAM files including all consensus reads, including off-target, were used as input. Based on the results from the variant calling of SNVs and InDels, 45 samples with negative mutation status (i.e. classified as ctDNA negative samples), and no evidence of CNAs, were used for construction of the reference file (Supplementary Table S10).

### Calculations of ctDNA fraction and absolute tumor burden

To estimate the fraction of cfDNA shredded from tumor cells ($\:c{tDNA}_{frac}$), we utilized the maximum non-amplified variant allele frequency (MAF). In addition, to compensate for any CNAs affecting the mutation allele frequency, e.g. loss of heterozygosity, the allelic composition of the variant locus was extracted from the visual overview produced by Jumble and included in the following formula:

1 $$\:\begin{array}{c}{ctDNA}_{frac}=\:\frac{2}{\frac{M}{MAF}\:-CN+2}\end{array}$$

where $\:CN$ is the total number of alleles and $\:M$ is the number of mutated alleles. The calculated $\:{ctDNA}_{frac}$ was derived from the equation below (2) as earlier described^37^, where $\:MSF$ is the mutated sample fraction, assuming clonal mutation and no CNA heterogeneity. Hence, in Eq. (1), $\:MSF{=ctDNA}_{frac}$. Moreover, whenever no copy number data was available, due to insufficient ctDNA fraction, the copy number background was assumed disomic ($\:CN=2$) with one mutated allele ($\:M=1$).

2 $$\:\begin{array}{c}MSF=\:\frac{VAF\bullet\:\left(CN*{ctDNA}_{frac}+2*\left(1-{ctDNA}_{frac}\right)\right)}{M}\end{array}$$

To account for varying levels of cfDNA in the blood from non-tumor cells, and their effect on the tumor burden assessment, we further normalized the ctDNA fraction against the total concentration of cfDNA (ng/ml plasma), and estimated the absolute number of tumor-derived DNA molecules, mutated genome equivalents (mGEs), as described^45^. This was calculated as the number of mGEs from the tumor per ml plasma, assuming 6 pg per diploid genome, as follows:

3 $$\:\begin{array}{c}\text{N}\text{u}\text{m}\text{b}\text{e}\text{r}\text{}\:\text{o}\text{f}\text{}\:\text{m}\text{G}\text{E}\text{s}\text{/}\text{m}\text{l}\:\text{}\text{p}\text{l}\text{a}\text{s}\text{m}\text{a}=\:\frac{{ctDNA}_{frac\:}\times\:\:{cfDNA\:}_{conc}\:\times\:\:1000}{6\:}\end{array}$$

For calculations of ctDNA levels, five cfDNA samples with negative ctDNA status were excluded due to a different analytical sequencing sensitivity, four were excluded due to usage of a broader sequencing panel in the pilot experiment (M05_2, M09_1_B, M09_3 and M14_2, see Methods), and one was excluded due to a decreased mean collapsed coverage (M11_2). A detailed description of all plasma samples is provided in Supplementary Table S1.

### Pilot experiment

A number of FFPE tissue (*n* = 25) and plasma (*n* = 16) samples, originating from the first 10 patients in the CHAMP-study with both longitudinal plasma samples and matching tumor tissue available, were part of an initial pilot experiment. These samples were analyzed at Eurofins Genomics (Konstanz, Germany), using their commercially available targeted sequencing solutions, see Supplementary Table S1. Eleven out of the 16 plasma samples were however re-sequenced using the customized CHAMP panel. For the tissue samples, 200 ng of genomic DNA was used for library preparation and enrichment with Agilent SureSelectXT Human All Exon V6 kit (Agilent), followed by paired-end (2 × 150 bp) targeted sequencing using the INVIEW Oncoprofiling panel (591 genes, sensitivity 1%) on the NovaSeq 6000 platform (Illumina). For the plasma samples, the second centrifugation, performed at 3250 *g* for 20 min after storage in -80 °C, cfDNA isolation and sequencing was done by Eurofins. cfDNA extraction was performed using the QIAamp Circulating Nucleic Acid kit, eluted in 34 µl buffer AVE (Qiagen), followed by concentration measurements and fragment size quality assessment. Thereafter, library preparation was performed on 100 ng cfDNA with Agilent SureSelect hybridization technology, followed by paired-end (2 × 150 bp) targeted sequencing using the INVIEW Liquid Biopsy Oncoprofiling panel (591 genes) on the NovaSeq 6000 instrument, aiming at a sequencing depth of 25 M read pairs per sample (sensitivity 1%).

To make the results comparable to the larger batch tissue and plasma samples, the subsequent bioinformatic analysis was performed in-house. Raw reads were trimmed for low quality and adaptor sequences using fastp (v 0.23.4)^46^ followed by alignment to the GRCh37 reference genome using BWA MEM. Variant calling was performed in regions covered in GMCK panel using freebayes and vardict-java, both with a variant frequency cutoff above or equal to 3%. Additionally, for variants called by freebayes only those with a ratio of mean quality between reference and alternate bases above or equal to 0.6 were kept. Variants were then decomposed, normalized, merged and annotated with VEP as described above. Further filtering was performed to only retain variants with a minimum depth of 300x and a variant frequency ratio between tumor and normal samples of above or equal to 2. Of note, for tumor tissue samples originating from patient M14, M16 and M18, adjacent normal tissue was used as germline control. For downstream analyses, only variants with allele frequencies of 10% or higher, or 4% or higher for gene regions included in the smaller CHAMP panel, and called by both callers, were kept. However, for tumor samples originating from patient M18, no mutations were detected with frequencies above 10% and, hence, only hotspot variants in *KRAS* were reported.

### Statistics

Statistical analyses were performed using R version 4.2.2. (Vienna, Austria). Fisher’s exact test was performed to investigate associations between proportions of categorical variables. Wilcoxon signed rank-test was performed to compare the medians of a continuous variable in two groups. Kaplan-Meier survival analysis was performed to assess associations between two groups and OS, using log rank test to calculate p-values. Cox univariable and multivariable regression was applied to calculate hazard ratios (HR) with 95% confidence intervals (CI) for death. To find the optimal prognostic cutoff value for ctDNA levels in the palliative patients, the function *surv_cutpoint* from the R package *survminer* was used, which implements maximally selected rank statistics from the R package *Maxstat*. The significance level was set to 0.05 for all statistical tests.

## Results

### Sample overview, patient demography and ctDNA status

**Fig. 1:**
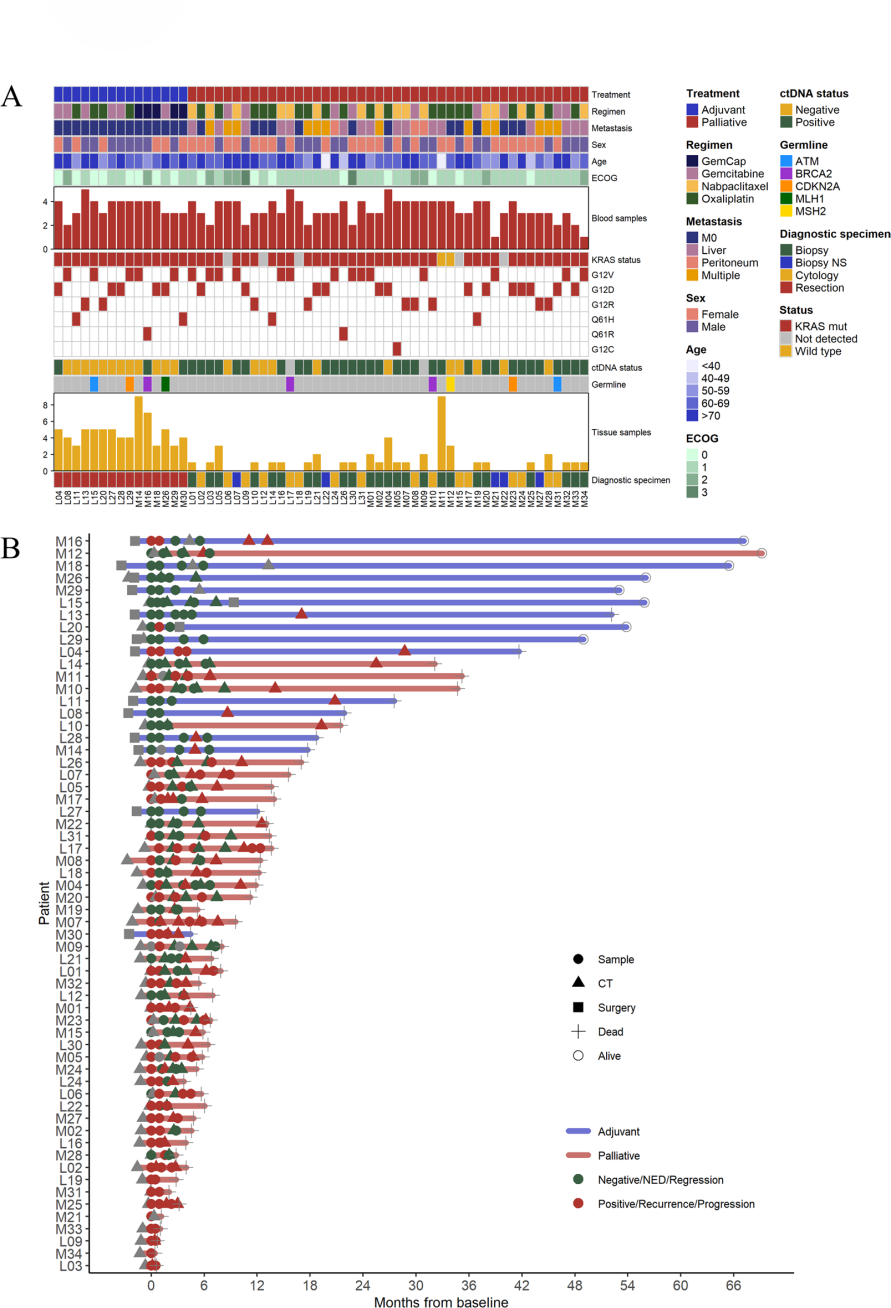
Demographic and clinical characteristics and sample overview for each patient. (**A**) Adjuvant vs. palliative treatment, chemotherapy regimen, distant metastasis (M0, liver, peritoneum or multiple sites), sex, age category and performance status according to the Eastern Cooperative Oncology Group (ECOG) scale, per patient. Red bars indicate the total number of analyzed plasma samples per patient followed by *KRAS* mutation status; mutated vs. wild-type or not detected, type of mutation, overall ctDNA status regardless of time point, germline mutation (if detected), yellow bars indicate the total number of analyzed tissue samples per patient followed by type of diagnostic specimen: surgical resection, biopsy, cytology, or biopsy not sufficient (NS) for analysis. (**B**) Overview of timepoints for ctDNA samples, CT scans and surgery for adjuvant (blue lines) and palliative (red lines) treated patients. Green depicts negative for ctDNA, no evidence of disease (NED) or radiologic regression, red depicts positive for ctDNA, recurrence or radiologic progression. Cross marks timepoint for death and circle means that the patient was alive at last follow-up.

A summary of the number of blood and tissue samples analyzed for each patient, overall *KRAS* mutation status, treatment regimen, germline variant information, and other clinical data are shown in Fig. 1A. Additional information on adjuvant patients is available in Supplementary Table S11. Fifteen patients (25%) had resectable tumors and received adjuvant chemotherapy, and 45 patients (75%) were treated with palliative intent. Of the resected tumors, 13 originated in the pancreas, one in the Ampulla of Vater (M16), with pancreatobiliary morphology, and one in the distal bile duct (M29), with pancreatobiliary morphology. All palliative patients had tumors deemed to originate in the pancreas. The vast majority, 96% (53/55) of the patients with assessable *KRAS* mutation status, had *KRAS* mutated tumors and two patients had *KRAS* wild-type tumors. In five patients, *KRAS* mutation status could not be assessed due to low ctDNA/tumor fractions. Following broad targeted NGS, pathogenic germline variants were detected in nine patients, three of whom had *BRCA2* mutations and two had mutations in mismatch repair genes *MLH1* and *MSH2*, further detailed in Supplementary Table S12.

In a total of 201 serial plasma samples (range 1–5, median = 3 per patient), ctDNA fluctuations were analyzed using the CHAMP panel (196/201) or targeted broad sequencing (5/201). Genetic data from matched tumor tissue was also included for 34 patients, with a median of one sample per patient (range 1–9) and 115 samples in total, after exclusion of 26 samples due to insufficient tumor cell content and one sample which failed sequencing (Supplementary Table S2 and S13).

Time points for blood sampling and disease trajectories, including radiologic workup and date of surgery, are shown in Fig. 1B. In adjuvant patients (*n* = 15), median OS was 44.4 months (range 11.3–61.4 months), and a total of 54 plasma samples were analyzed (median 4 samples, range 2–5, per patient). In palliative patients (*n* = 45), median OS was 7.6 months (range 1.1–36.3 months), excluding patient M12 who had Lynch syndrome, received immunotherapy in later lines and is still alive after more than five years, and a total of 147 plasma samples were examined (median 3 samples, range 1–5, per patient). In two patients, L15 and L20, blood sampling was only performed during neoadjuvant treatment.

As shown in Supplementary Figure S1, BL levels of routine clinical biomarkers CA19-9, carcinoembryonic antigen (CEA), c-reactive protein (CRP), but not albumin, were significantly higher in palliative than in adjuvant patients.

### Distribution and clinicopathological correlates of ctDNA levels and identification of a robust prognostic cutoff in palliative patients

**Fig. 2:**
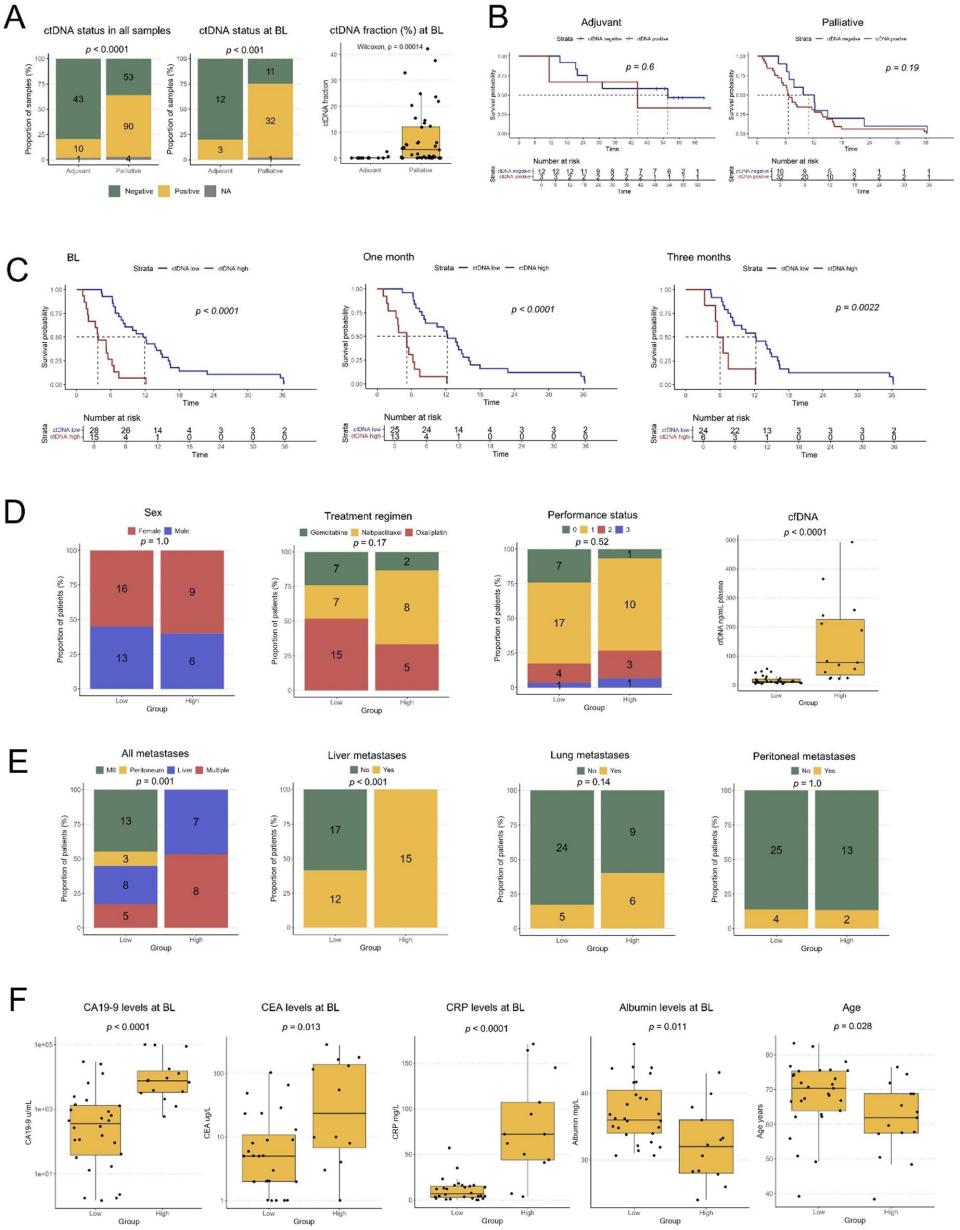
Clinicopathological correlates and prognostic impact of ctDNA levels. (**A**) Total number of ctDNA positive and negative plasma samples during treatment (left panel), ctDNA status for each patient at baseline (middle panel), and ctDNA fractions at baseline (right panel), in adjuvant and palliative patients, respectively. NA marks samples with a different, and less sensitive, detection limit. BL = baseline. (**B**) Kaplan-Meier analysis of overall survival (OS) in strata according to positive vs. negative ctDNA status at baseline in adjuvant patients (left panel) and in palliative patients (right panel). (**C**) Kaplan-Meier analysis of OS in palliative patients in strata according to high (ctDNA^high^) vs. low (ctDNA^low^) ctDNA concentrations at baseline, one month and three months of treatment, defined by the calculated optimal prognostic cutoff value at 350 mGEs/ml plasma. (**D**) From left to right: Distribution of sex, treatment regimen, ECOG performance status and cfDNA levels in ctDNA^low^ and ctDNA^high^ patients, respectively. (**E**) From left to right: Distribution of metastatic status and metastatic sites, liver metastases, lung metastases and peritoneal metastases in the ctDNA^low^ and ctDNA^high^ groups, respectively. For (**D–E**) Fisher’s exact test was used to calculate associations between proportions of categorical variables. (**F**) Levels of routine clinical parameters CA19-9, CEA, CRP and albumin, and distribution of age in ctDNA^low^ and ctDNA^high^ patients, respectively.

The total number of ctDNA positive samples during treatment, the number of patients with ctDNA positive samples at BL, and the ctDNA fraction at BL in adjuvant and palliative patients, respectively, are shown in Fig. 2A.

In line with the expected, the total number of ctDNA positive samples during treatment differed significantly between adjuvant (10/53, 19%) and palliative (90/143, 63%) patients. In adjuvant patients, the median ctDNA fraction at BL was 0% (range 0-2.43%), with three patients (20%) having a positive ctDNA sample before starting treatment. In palliative patients, the median ctDNA fraction at BL was 3.2% (range 0-42.1%), with 74% (32/43) having detectable ctDNA in their blood before starting treatment.

As shown in Fig. 2B, positive vs. negative ctDNA status at BL was not significantly associated with OS in either patient group. However, as the ctDNA fraction is influenced by fluctuations in the total amount of cfDNA, we instead calculated the absolute number of mGEs per ml plasma as a proxy for tumor burden^45,47,48^. As a proof of concept, the varying levels of ctDNA fraction, total cfDNA concentration and number of mGEs, respectively, are shown in Supplementary Figure S2.

Moving forward with the mGE measurements in palliative patients, we assessed their potential prognostic value by performing univariable Cox regression analysis in continuous units of 1000 mGE/ml plasma, at BL. This measurement was prognostic both in univariable analysis (HR = 1.2, 95% CI = 1.1–1.3, *p* < 0.0001) and in multivariable analysis, adjusted for age, performance status, disease stage (M0/M1), and regimen (HR = 1.2, 95% CI = 1.1–1.3, *p* < 0.001). Next, an optimal prognostic cutoff value was identified at 350 mGE/ml plasma, whereby patients with plasma levels below and above this threshold, respectively, at BL were classified as ctDNA^low^ (*n* = 28) or ctDNA^high^ (*n* = 15), excluding patient L17 (no BL sample) and patient M12 (Lynch syndrome). Patient M09 had a negative ctDNA at BL using the broader and less sensitive panel and was therefore not included in the calculation of the optimal prognostic mGE value, but was later assigned to the ctDNA^low^ group. As shown in the Kaplan-Meier curves in Fig. 2C, patients in the ctDNA^high^ group had a significantly reduced OS compared to patients in the ctDNA^low^ group not only at BL (median 3.7 versus 11.9 months), but also at 1M (5.16 versus 12.3 months) and 3M (6.01 versus 12.2 months). Notably, in the ctDNA^low^ group, about one out of three patients (*n* = 11) had a ctDNA negative sample at BL.

Next, we compared the distribution of relevant patient and tumor characteristics between the ctDNA^low^ and ctDNA^high^ groups. As shown in Fig. 2D, there were no significant differences regarding sex, treatment regimen, or performance status. However, in line with the expected, patients in the ctDNA^high^ group had a significantly higher concentration of cfDNA in their blood at BL compared to the ctDNA^low^ group (77.9 versus 12.0 ng/ml). As further shown in Fig. 2E, all patients in the ctDNA^high^ group had metastatic disease at diagnosis, with dissemination either to the liver only (*n* = 7), or to multiple sites including the liver (*n* = 8). In contrast, 55% (*n* = 16) of the patients in the ctDNA^low^ group had metastatic disease at diagnosis, eight with liver metastases only, three with peritoneal metastases only, and five with metastases at multiple sites. Only a few patients in each group had lung metastases, with no significant difference, and only in combination with metastases at other sites. Lastly, as shown in Fig. 2F, levels of routine biomarkers, CA19-9, CEA and CRP were significantly higher in the ctDNA^high^ group at BL, although with a relatively high variability for CEA within the groups, whereas albumin levels were significantly lower in the ctDNA^high^ group at BL. Differences in age at diagnosis were also observed, with a median of 61.8 years in the ctDNA^high^ group compared to 70.3 years in the ctDNA^low^ group.

**Fig. 3:**
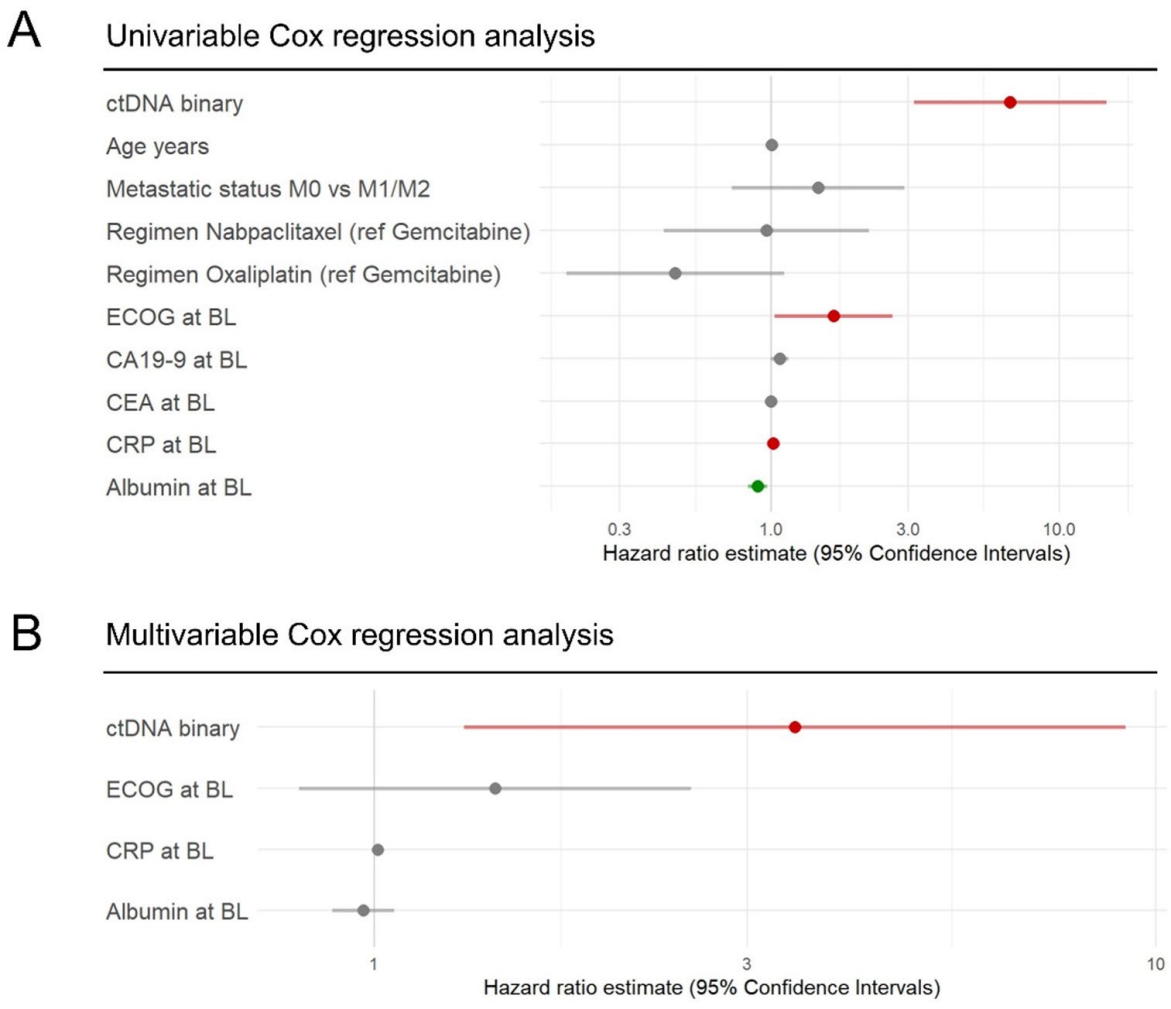
Prognostic value of ctDNA and clinical parameters in palliative patients. (**A**) Hazard ratio for death with 95% confidence intervals from univariable Cox regression analysis of binary ctDNA stratification into low vs. high concentration at baseline, age at diagnosis in years, metastatic status (M0 vs. M1/M2), regimen Nabpaclitaxel and Oxaliplatin against reference gemcitabine, respectively, performance status according to the Eastern Cooperative Oncology Group (ECOG) scale^63^ (levels 2 and 3 combined) and clinical routine parameters CA19-9, CEA, CRP and albumin, respectively. (**B**) Hazard ratio for death with 95% confidence intervals from multivariable Cox regression analysis of binary ctDNA stratification into low vs. high concentration at baseline adjusted for performance status according to the ECOG scale, CRP and albumin levels at baseline.

The prognostic value of the binary ctDNA stratification at BL in the palliative patient group was further confirmed in univariable Cox regression analysis (Fig. 3A). Among established clinical parameters, performance status, CRP and albumin levels were also prognostic in univariable analysis. After adjustment for these parameters in multivariable analysis (Fig. 3B), ctDNA stratification remained the sole independent prognostic factor (HR = 3.5, 95% CI = 1.3–9.1).

These results highlight the potential utility of upfront, pre-treatment designation of a high and low ctDNA category, based on the absolute number of mGEs per ml plasma, for reliable prognostication over time, even outperforming established clinical parameters.

### CtDNA trajectories during chemotherapy and their relationship to the disease dynamics

**Fig. 4:**
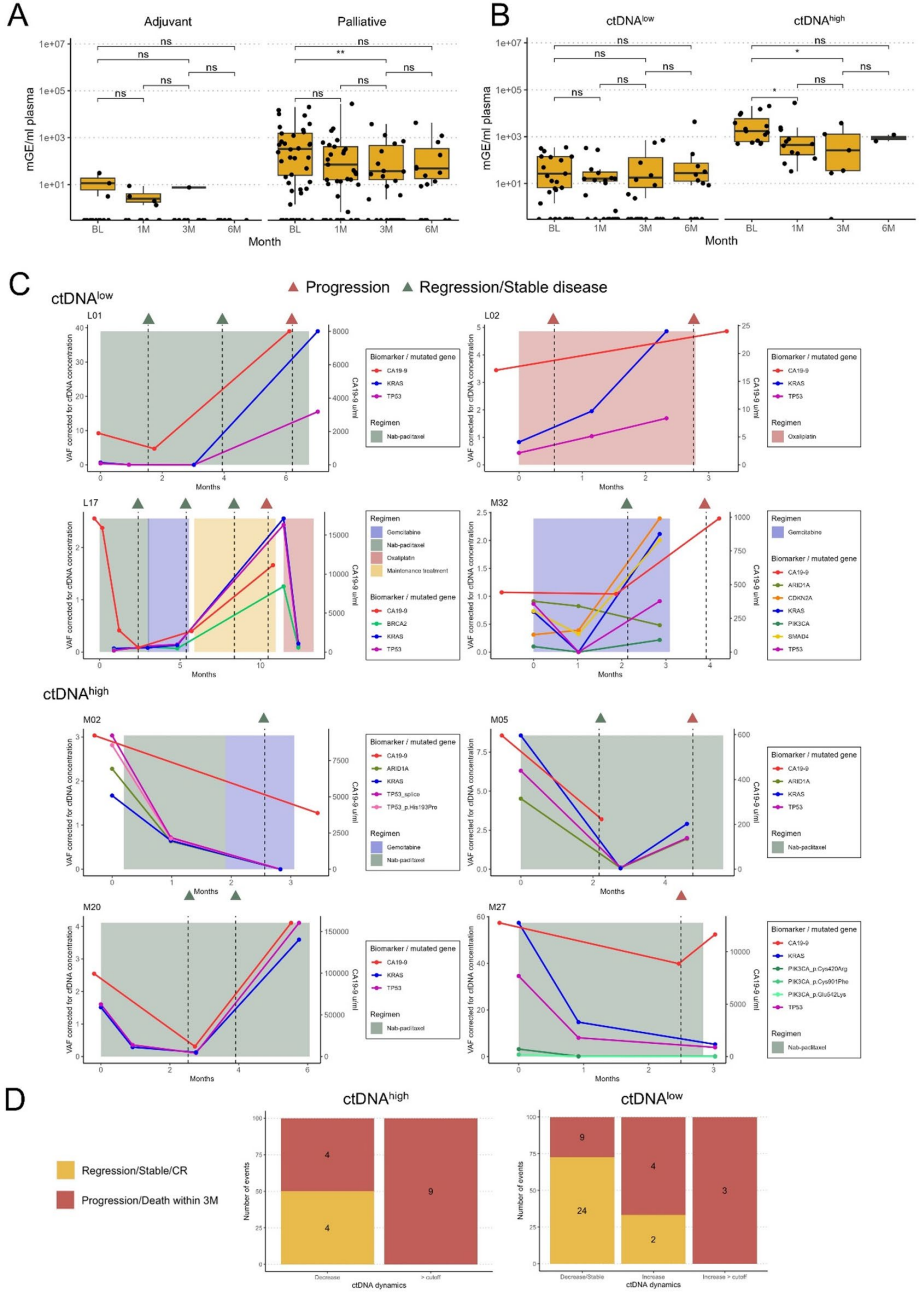
ctDNA dynamics during chemotherapy in relation to disease course, radiology reports and levels of CA19-9. (**A**) Levels of ctDNA (mGE/ml plasma) at different timepoints during treatment for adjuvant and palliative patients, respectively, and (**B**) for palliative patients in the ctDNA^low^ and ctDNA^high^ group, respectively (ns = not significant, * = *p* < 0.05, ** *p* < 0.01, in Wilcoxon sign-rank test). (**C**) Trajectories for cfDNA corrected variant allele frequencies (VAF) of gene variants and levels of CA19-9 in plasma for eight selected patients, three in the ctDNA^low^, four in the ctDNA^high^ group and patient L17, together with treatment regimens and radiological evaluations (green triangle = radiological regression or stable disease, red triangle = radiological progression). (**D**) Evaluation of changes in ctDNA (Δ-ctDNA) related to disease status at any clinical workup, during treatment or at the end of treatment, in the ctDNA^high^ and ctDNA^low^ patient groups, respectively. In the ctDNA^high^ group, Δ-ctDNA categories were defined as a decrease, if the resulting ctDNA concentration was below the prognostic cutoff (350 mGEs/ml plasma), or as staying above the cutoff, regardless of Δ-ctDNA. In the ctDNA^low^ group, Δ-ctDNA was defined as a decrease if the Δ-ctDNA was less than 25 units (mGEs/ml plasma), stable if the change was within 25 absolute units, and an increase as a change that was greater than 25 units, either below or above the prognostic cutoff value.

We also set out to evaluate the potential value of ctDNA as a tool for on-treatment monitoring of the disease dynamics. As shown in Fig. 4A, there was an overall decline in ctDNA levels during chemotherapy between BL and 3M in palliative patients. No significant changes were detected in adjuvant patients. Further comparison of the ctDNA^low^ and ctDNA^high^ groups in palliative patients (Fig. 4B) showed that the observed decline in ctDNA levels was primarily due to changes in the ctDNA^high^ group, with a significant decrease between both BL and 1M and BL and 3M. These changes did however not remain significant after Bonferroni correction for multiple testing.

Next, we explored the potential clinical utility of patient specific ctDNA trajectories within the ctDNA^low^ and ctDNA^high^ patient groups. On-treatment trajectories of blood derived gene variants are shown in Fig. 4C for eight selected patients, four in the ctDNA^high^ group with samples from at least three time points and the three patients in the ctDNA^low^ group where ctDNA levels rose above the ctDNA^high^ cutoff value during, or after, treatment, along with patient L17, for whom no BL sample was available but who had a particularly fluctuating trajectory. Trajectories for the remaining patients can be found in Supplementary Figure S3 (details in Supplementary Table S14). Overall, VAF values of different variants fluctuated in tandem over time. However, for patient M32, the longitudinal pattern of a mutation detected in the *ARID1A* gene, and lower VAFs for a variant detected in the *PIK3CA* gene, deviated from the dynamics of the rest of the detected variants, showing possible signs of temporal heterogeneity. ctDNA trajectories were also compared to levels of CA19-9 and the clinical disease course. Although CA19-9 was not measured as frequently as ctDNA, and not at the same timepoints, there was a weak positive correlation between the slopes of the first two measurements (Spearman’s correlation coefficient = 0.38).

Focusing on the ctDNA^low^ group, increased levels of ctDNA during treatment (above > 350 mGE/ml) were associated with progressive disease in all four cases. On the other hand, in the ctDNA^high^ group, longitudinal monitoring of ctDNA displayed less pronounced concordance to the clinical disease course.

To further investigate our observations on ctDNA dynamics and disease progression in the two groups, we compared all ctDNA changes (Δ-ctDNA) associated with disease status at each clinical workup during and at the end of treatment. For this analysis, we included all patients with a minimum of two assessable plasma samples, with at least one being ctDNA positive (34 patients, 59 Δ-ctDNA events, Supplementary Table S15), as seen in Fig. 4D. For patients in the ctDNA^high^ group, a decrease resulting in ctDNA levels below the prognostic cutoff value was only associated with disease regression or stable disease for four out of eight (50%) Δ-ctDNA events. For the four Δ-ctDNA events associated with disease regression or stable disease, the median percentage of decrease was 95,5%, and for the four Δ-ctDNA events associated with disease progression or death within three months, the median percentage of decrease was 63,0%. Furthermore, the dynamics of ctDNA did not have any significant impact on disease outcome as long as the total ctDNA level remained above the cutoff value, as all Δ-ctDNA events were associated with progression or death within three months. On the other hand, for the ctDNA^low^ group, decreased or stable ctDNA levels were most often linked to regression or stable disease (24/33 Δ-ctDNA events, 73%). Notably, an increase in ctDNA levels above the prognostic cutoff was consistently correlated to disease progression (3/3 Δ-ctDNA events).

These results suggest that monitoring of ctDNA levels more accurately predicts disease dynamics in palliative patients upfront assigned to the ctDNA^low^ group, compared to the ctDNA^high^ group. Additionally, they validate the utility of the mGE based cutoff for prognostication.

### The mutational landscape of ctDNA captures and complements tumor tissue analyses and holds potential as a tool for precision medicine

**Fig. 5:**
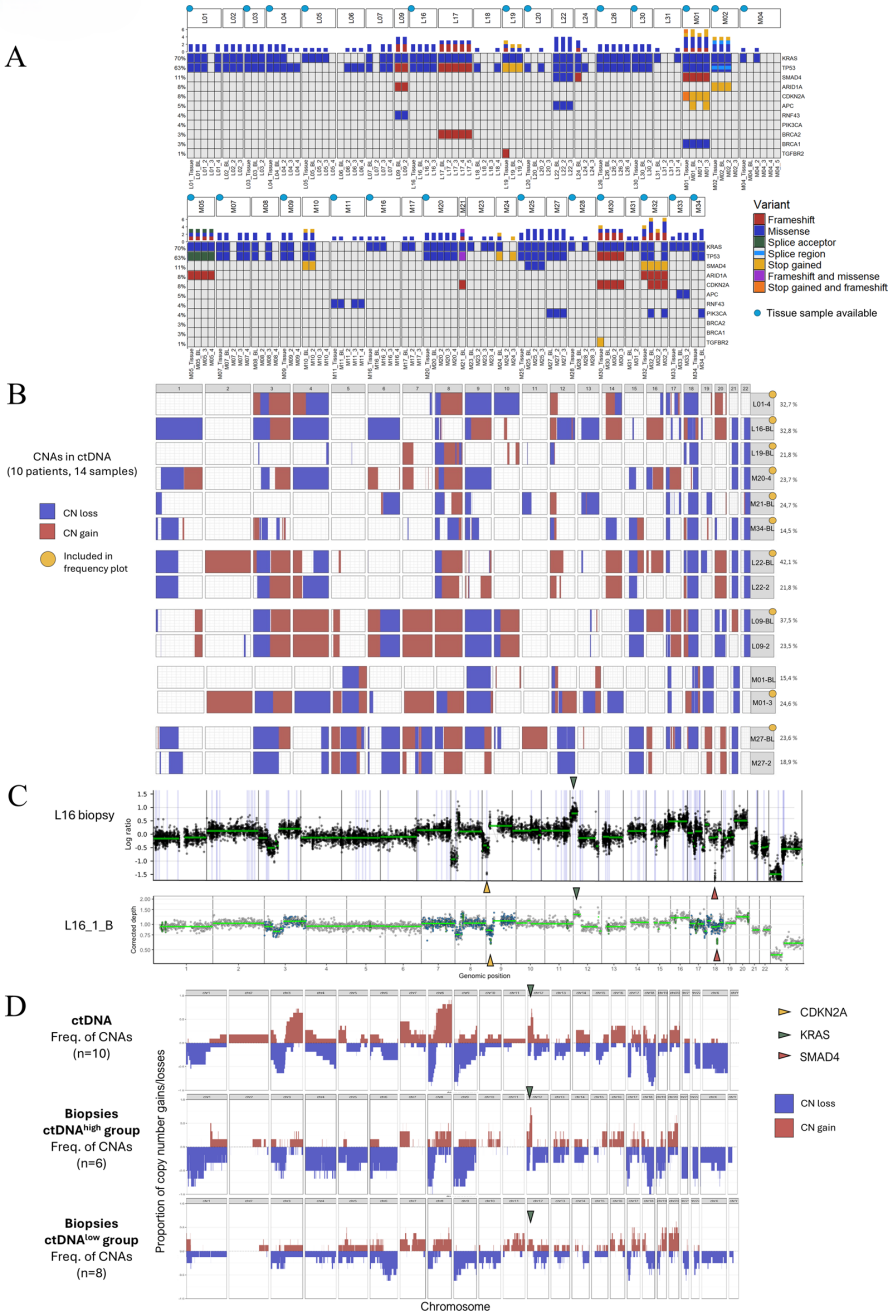
ctDNA and tissue derived somatic variants and copy number profiles. (**A**) Somatic variants detected in ctDNA and matched tumor tissue samples whenever available. Of note, patient M27 had three different missense variants in the *PIK3CA* gene. (**B**) Detected copy number changes (gains and losses with a minimum sample fraction of 10%), in chromosomes 1–22, for ten patients and a total of 14 samples with ctDNA fractions above 14.5%. Gains are shown in red and losses in blue. For four patients, two longitudinal samples are shown. Samples included in the frequency plot below are marked in yellow. (**C**) Comparison of the copy number profile in ctDNA and a matched diagnostic biopsy for patient L16. A 12p amplification covering *KRAS*, and homozygous deletions covering *CDKN2A* and *SMAD4* on 17p and 18q, respectively, are annotated. In the ctDNA panel, target bins are colored based on; exonic regions = green, SNP positions = blue, off-target = grey. (**D**) Summarizing frequency plots of gains and losses (one sample per patient, minimum 10% sample fraction) in ctDNA (*n* = 10) and in tumor tissue originating from the ctDNA^high^ (*n* = 6) and ctDNA^low^ (*n* = 8) patient groups respectively. Gains covering the *KRAS* locus in each graph are noted in green.

To evaluate genetic intrapatient heterogeneity, both temporally between longitudinal ctDNA samples (41 patients, 90 samples) and to matched tumor tissue whenever available (24 patients, 57 samples), we compared all detected variants found in genes included in the CHAMP-panel for all patients with at least one ctDNA positive sample (Fig. 5A). Overall, the most frequently mutated genes were *KRAS* and *TP53*, followed by *SMAD4* and *CDKN2A*. Notably, no intrapatient temporal heterogeneity was detected between plasma samples from different time points, after omitting all disparities that could not be ruled out as a consequence of reduced ctDNA abundance. Comparisons of the mutational landscape between matched cfDNA and tumor samples showed discrepancies in seven cases (L19, M01, M25, M30, M32, M33, M34), considering patients with at least one plasma sample with a ctDNA fraction above 2%. For instance, a stop gained variant was detected in the *APC* gene in plasma samples from M01 and a missense variant was detected in *SMAD4* in plasma samples from patient M25, neither of which were detected in matched tumor tissue. On the contrary, in L19 and M30, a frameshift variant and a stop gained variant, respectively, were detected in the *TGFBR2* gene in the matched tumor tissue, but not in any of the plasma samples. Of note, the *TGFBR2* variant in M30 was only detected in one out of four tumor samples.

The customized ctDNA panel was also designed to enable detection of common targets for precision medicine, approved for other solid cancers, which would be especially important for cases with no available tissue sample (47% of the palliative patients in the present study). For instance, a *KRAS*^*G12C*^ mutation was found in patient M05, meeting the criteria for targeted treatment with the selective KRAS-G12C inhibitor sotorasib. In addition, a somatic mutation in *BRCA1* was detected in patient M01 and two *BRCA2* variants, one germline and one somatic, were found in patient L17, for which there are targeted treatment options available, such as the PARP-inhibitor olaparib. Finally, mutations in *PIK3CA* were exclusively detected in plasma samples in three patients (M27, M32 and M34), where PI3K inhibitors such as alpelisib could be considered a treatment option.

These results demonstrate that the herein applied customized ctDNA panel allows for identification of common targets for personalized medicine with equivalent, and potentially even better, precision than tumor tissue analyses.

### Copy number profiles in ctDNA align well with matched tumor tissue and provide additional information on the temporal genetic heterogeneity during chemotherapy

In ten of the palliative patients, and a total of 14 samples, we were able to detect plasma derived copy number profiles across the whole genome (Fig. 5B). The ctDNA fractions for these samples were estimated to a median of 23.6% (range 14.5–42.1%). Moreover, in an additional ten patients, and 19 samples, SNP data could also aid estimation of ctDNA fractions by providing information on underlying allelic imbalances and amplifications. Cumulatively, by combining b-allele frequency (BAF) data from the incorporated SNPs, along with log_2_-ratios derived from all condensed reads ranging over the entire genome (including off-target reads), integration of copy number data from plasma derived ctDNA provided valuable information in 47% (*n* = 20) of the palliative patients (for details, see Supplementary Table S1). Discrepancies between the unmodified and the CNA corrected MAF based ctDNA fractions are shown in Supplementary Figure S4, emphasizing the advantage of CNA incorporation for ctDNA monitoring over time. Moreover, amplified or duplicated regions including the *KRAS* and *MYC* genes were detected in ctDNA from twelve and eleven patients, respectively.

To further validate the detected plasma-derived copy number profiles (*n* = 10), we compared them to matched tumor tissue whenever possible, considering tissue availability and sufficient tumor cell fractions (*n* = 5), as shown in Supplementary Figure S4. Overall, the copy number profiles from matched tissue and ctDNA samples conformed closely with each other, even for chromosomes not covered by SNPs, as demonstrated for patient L16 in Fig. 4C. For instance, an amplification including *KRAS* on chromosome 12 and homozygous losses of regions including *CDKN2A* and *SMAD4* on chromosome 9 and 18, respectively, were seen in both the diagnostic tumor biopsy and the BL plasma sample. On the other hand, a *KRAS* amplification detected after seven months of treatment in patient L01 was not found in the matched treatment naïve tumor biopsy, or in the BL plasma sample (Supplementary Figure S5). However, for the BL sample, we could not rule out the absence to be just an effect of a lower ctDNA plasma fraction (5%).

Moreover, in Fig. 5D, a summarizing graph of CNA frequencies throughout the genome for plasma samples with sufficient ctDNA fractions (*n* = 10), and tissue samples for the ctDNA^high^ (*n* = 6) and ctDNA^low^ (*n* = 8) group, are shown separately. In general, similar frequency patterns were seen for ctDNA and tumor tissue derived CNAs, with for instance loss of 17p and 18q in a large proportion of the samples in both groups. Furthermore, ctDNA samples and tumor samples in the ctDNA^high^ group showed a trend towards higher prevalence of 12p chromosomal gains (including *KRAS*) compared to the ctDNA^low^ group. For sample specific copy number profiles from different areas of each resected tumor in adjuvant patients and in one autopsied case (M11), see Supplementary Figure S6.

These results demonstrate the feasibility of CNA assessment in ctDNA, using ultra-deep targeted sequencing, and its advantage for a more accurate estimation of the ctDNA fraction. Moreover, the results further promote the utilization of CNA analyses to gain additional insight into treatment-driven genetic evolution and development of resistance.

## Discussion

In this study, we have compiled a comprehensive amount of real-world data from an observational clinical study in the attempt to create a blueprint of qualitative and quantitative changes in ctDNA during chemotherapy treatment in patients newly diagnosed with pancreatic cancer. Particular focus was placed on palliative patients receiving first-line chemotherapy, as they have an overall higher ctDNA burden than adjuvant patients, have no prospect of cure and make up the vast majority of patients.

We started out by examining the prognostic value of ctDNA status prior to the initiation of chemotherapy treatment, and contrary to a number of previous studies on patients with advanced pancreatic cancer^7,10,15^, stratification into ctDNA positive and negative categories was not prognostic in our cohort. Several factors may contribute to this discrepancy, including sample size, differences in sensitivity of the methods and number and size of target regions. Worth noting, however, is that the proportion of negative cases in the palliative patient group was smaller than in most other studies. Therefore, we cannot claim that a more sensitive detection method, such as ddPCR^7^, would have made ctDNA positivity vs. negativity a better prognostic indicator for this patient group. Of note, by utilizing our customized gene panel, we were able to detect ctDNA even in patients with *KRAS* wild-type tumors. On the contrary, for patients with resectable disease, the number of cases with positive ctDNA baseline status was lower than in previous research^49,50^. This is most probably due to the differences in assay sensitivity between a tumor-informed approach compared to the tumor-naïve approach utilized in this study, the latter addressing the issue of tumor tissue limitations in advanced disease.

A few previous studies have proposed that a more quantitative approach may offer further refined subgroups within palliative patients^6,7^, although still without any consensus on a defined prognostic cutoff. For example, in a study by Patel et al., the patients were divided by the median total fraction of ctDNA, following NGS, whereby patients with ctDNA levels above 0.6% had a worse prognosis (11.7 versus 6.3 months)^6^. However, as the ctDNA fraction remains dependent on the total cfDNA concentration at any given time point, the absolute number of mutated ctDNA molecules per ml plasma has instead been suggested to be a better proxy for the circulating tumor burden^51^. On that note, Pietrazs et al. instead utilized ddPCR to group the patients into tertiles based on the absolute quantity of ctDNA, which also revealed a significant association between ctDNA quantity and survival^7^. However, challenges remain in comparing quantitative results from different technologies, such as ddPCR and NGS-based assays^5^, in a satisfactory manner.

Thus, we moved forward with calculations of absolute pre-treatment ctDNA levels in palliative patients, whereafter an optimized prognostic model was applied, with a median OS of 3.7 months in the ctDNA^high^ group, and a median OS of 11.9 months in the ctDNA^low^ group. Of note, although the detected optimal prognostic ctDNA cutoff was calculated from BL levels, it remained strongly significant in subsequent measurements at 1M and 3M. Moreover, and importantly, the calculated ctDNA cutoff remained the only independent prognostic factor in multivariable analysis.

To our best knowledge, no previous studies have assessed the prognostic utility of an optimized ctDNA-based cutoff in relation to absolute pre-treatment levels in plasma. Hence, we postulate that an upfront dichotomization into ctDNA^high^ and ctDNA^low^ groups could constitute a meaningful first step in a more tailored treatment approach, as the ctDNA^low^ group appears to give an adequate definition of palliative patients who are likely to have a comparatively good response to first line chemotherapy. Moreover, since the ctDNA dynamics was a more accurate indicator of disease progression and regression in the ctDNA^low^ group, we also postulate that on-treatment monitoring of ctDNA and/or other biomarkers could be of particular value in the ctDNA^low^ group, potentially to guide adaptive treatment strategies. Regarding the ctDNA^high^ group, consisting of fewer patients, it is evident that any window of opportunity to find an effective treatment strategy will only briefly be left ajar. Given their higher ctDNA levels, rapid upfront identification of targetable alterations could change the outlook for some of these patients. On the other hand, if no treatment targets can be identified, our results indicate that patients in the ctDNA^high^ group with rising or persistently high ctDNA levels, above the prognostic cutoff, after chemotherapy initiation will likely have little or no treatment benefit. Thus, for these patients, early initiation of best supportive care could increase their chances of having an increased quality of life in the few remaining weeks or months of life. It must however be pointed out that the herein calculated cutoff needs further validation in additional studies.

Of note, all patients in the CHAMP study are treated with chemotherapy, since this is an inclusion criterion, and one of the main aims is to study tumor evolution during chemotherapy. Thus, patients who are too ill to be eligible for chemotherapy are excluded. It is therefore possible that we may miss some potentially important information on the specific biology of their tumors, which is a potential weakness of the study. On the other hand, it can also be argued that the results from this article are based on real-world data from a group of patients who is sufficiently homogeneous for identification of clinically relevant patterns in the on-treatment disease dynamics, which may help predict the duration of response to chemotherapy and guide precision medicine. In line with this reasoning, it would not be meaningful to compare the results with a control group not receiving systemic chemotherapy.

Comparisons of the clinical characteristics between the groups showed that the prevalence of distant metastases was significantly higher in the ctDNA^high^ (100%) than in the ctDNA^low^ (60%) group, and comparisons by metastatic site revealed that this difference was only significant for liver, not lung or peritoneal, metastases. The finding that liver metastases contribute to higher levels of ctDNA in the circulation is in line with previous studies on PDAC and other types of cancer^7,52^. Moreover, levels of routine clinical blood parameters CEA, CA19-9 and CRP were, as expected, higher, and albumin was lower in the ctDNA^high^ group. The finding of ctDNA levels being inversely associated with age is also supported by previous research^7^. Age was however not prognostic in the present study cohort and existing literature supports that age, if anything, is linked to shorter survival^53^.

A secondary objective in the design of the CHAMP ctDNA panel was to facilitate plasma identification of alterations in 23 genes relevant for precision medicine, as this has provided clinically relevant information in other studies, even when utilizing smaller gene panels^3,54,55^. This is of particular importance in PDAC patients where tumor tissue is rarely available at diagnosis, which was the case for almost half of the palliative patients in our cohort, and even less so at disease progression, as repeat tissue biopsies are almost never obtained. Significant findings included somatic mutations in *KRAS* (G12C), *BRCA1/2* and *PIK3CA*, for which there are targeted treatments approved for other solid cancers. Worth noting is that the *PIK3CA* mutation was exclusively detected in blood. In addition, by broad targeted sequencing of normal blood, germline variants with neoplastic pathogenicity were detected in genes known to be tumor agnostic biomarkers predicting response to immunotherapy (*MLH1* and *MSH2*), or to PARP-inhibitors (*BRCA2* and *ATM*). These findings further support the importance of upfront molecular testing in all PDAC patients.

Whenever possible, we also set out to evaluate the extent of genetic heterogeneity between serial plasma samples as well as between plasma samples and, matched tumor tissue. While we could not detect any intrapatient genetic temporal heterogeneity, the mutational landscape between cfDNA and matched tumor samples showed discrepancies in seven patients, in that some mutations were seen only in ctDNA and others only in tissue, similarly to other studies^6,56^. This observed heterogeneity highlights the added value of liquid biopsies as a complement to single-lesion tumor biopsies, as these may not reflect the overall genetic landscape of the tumor^4,57,58^. In addition, the frequent lack of high-yield tissue samples, owing to the location of the primary tumor deep in the abdomen, and an overall sparse tumor cell fraction due to an often dominating desmoplastic component, underscores the importance of liquid biopsy implementation in PDAC. On the other hand, the copy number profiles from matched tissue and ctDNA samples showed a higher degree of similarity, validating our small customized ctDNA design for genome-wide copy number calling, consistent with another recent publication^59^. These results are also in line with a previous study utilizing shallow whole genome sequencing on longitudinal cfDNA samples in advanced PDAC^60^. An amplification covering the *KRAS* gene was, however, detected in a plasma sample associated with disease progression in one patient with a *KRAS* mutated tumor, which is also in line with previous research^61^. Speculatively, as the amplification was not detectable in the diagnostic pre-treatment biopsy, this could indicate signs of treatment resistance as previously discussed in a PDAC case report^62^.

In summary, these real-world data support the potential value of on-treatment ctDNA monitoring for assessment of response to chemotherapy in patients with locally advanced or metastatic pancreatic cancer. Accordingly, the identification of an appropriate prognostic cutoff at baseline depends on both the context and methodology, which must be considered in validatory studies. The results also demonstrate the versatile clinical utility of applying a customized and focused sequencing approach for ctDNA, that serves as a reliable non-invasive alternative to tumor tissue analyses over time, both regarding small variants and copy number profiles.

## Supplementary Information

Below is the link to the electronic supplementary material.

Supplementary Material 1

Supplementary Material 2

## Data Availability

Due to constraints related to patient privacy and institutional regulations, we cannot publicly share the source data files, sequencing data, and code used in this article. De-identified individual participant data, including data dictionaries and code, will be shared with researchers whose proposed use of the data is methodologically sound and in accordance with ethical and legal obligations. Proposals should be directed to the study principal investigator, email karin.jirstrom@med.lu.se . All other data supporting the findings of this study are available within the article and its supplementary files.
