# Supplementary material for: Reliable on-treatment prognostication and target identification with a customized assay for circulating tumor DNA in patients with newly diagnosed pancreatic cancer
Source: Sci Rep. 2025 Oct 3;15:34481. doi: 10.1038/s41598-025-22369-5 (PMC12494785; doi:10.1038/s41598-025-22369-5)
Supplement: Supplementary file 1 — Supplementary Material 1 [file 41598_2025_22369_MOESM1_ESM.pdf]

Supplementary Figures

Figure S1. Routine clinical parameters in adjuvant and palliative patients at baseline

Figure S2. Levels of ctDNA fraction, cfDNA concentration and mutated genome equivalents in all patients

Figure S3. Variant and CA19-9 trajectories of all ctDNA positive patients

Figure S4. CNA corrected ctDNA fraction comparison and CNAs in blood and tissue

Figure S5. Comparison of CNAs detected in matched ctDNA and tumor tissue samples

Figure S6. Multiregional copy number profiles

Figure S1

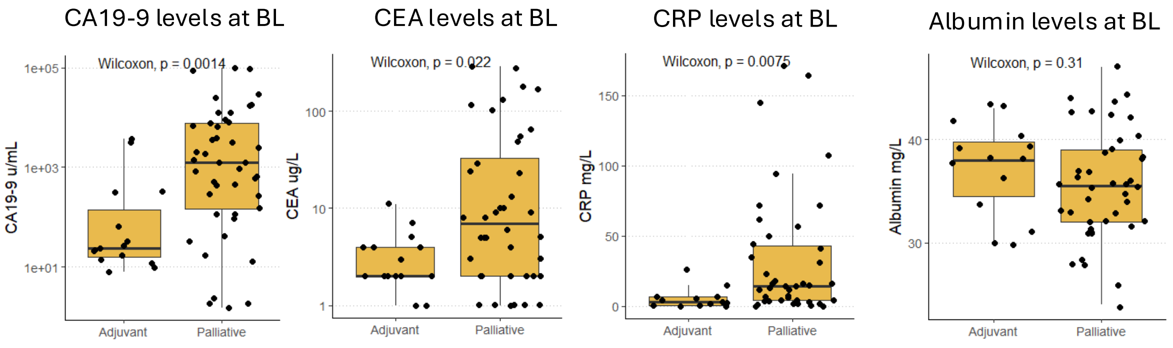

Figure S1. Levels of routine clinical parameters CA19-9, CEA, CRP and Albumin at baseline in adjuvant and palliative patients, respectively.

Figure S2

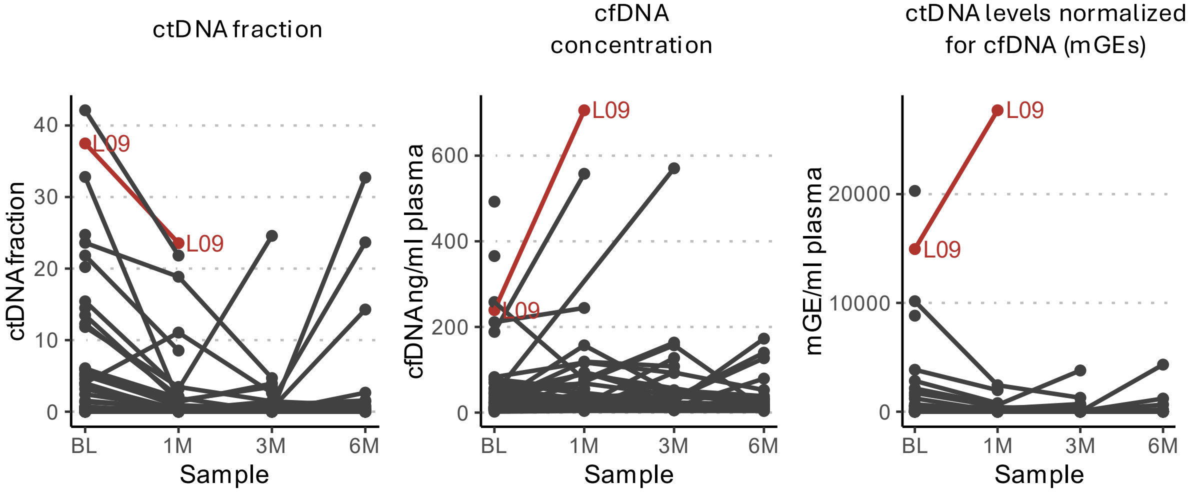

Figure S2. Levels of ctDNA fraction (left panel), total cfDNA concentration (middle panel) and the number of mutated genome equivalents (mGE) per ml plasma (right panel) in all patients over time. As an example, when examining samples from before and after one month of treatment in patient L09, the ctDNA fraction notably declines. However, when analyzing the total cfDNA concentrations at the same time points, a dramatic rise is denoted, and, hence, the absolute number of mGEs has actually increased between the two time points.

Figure S3

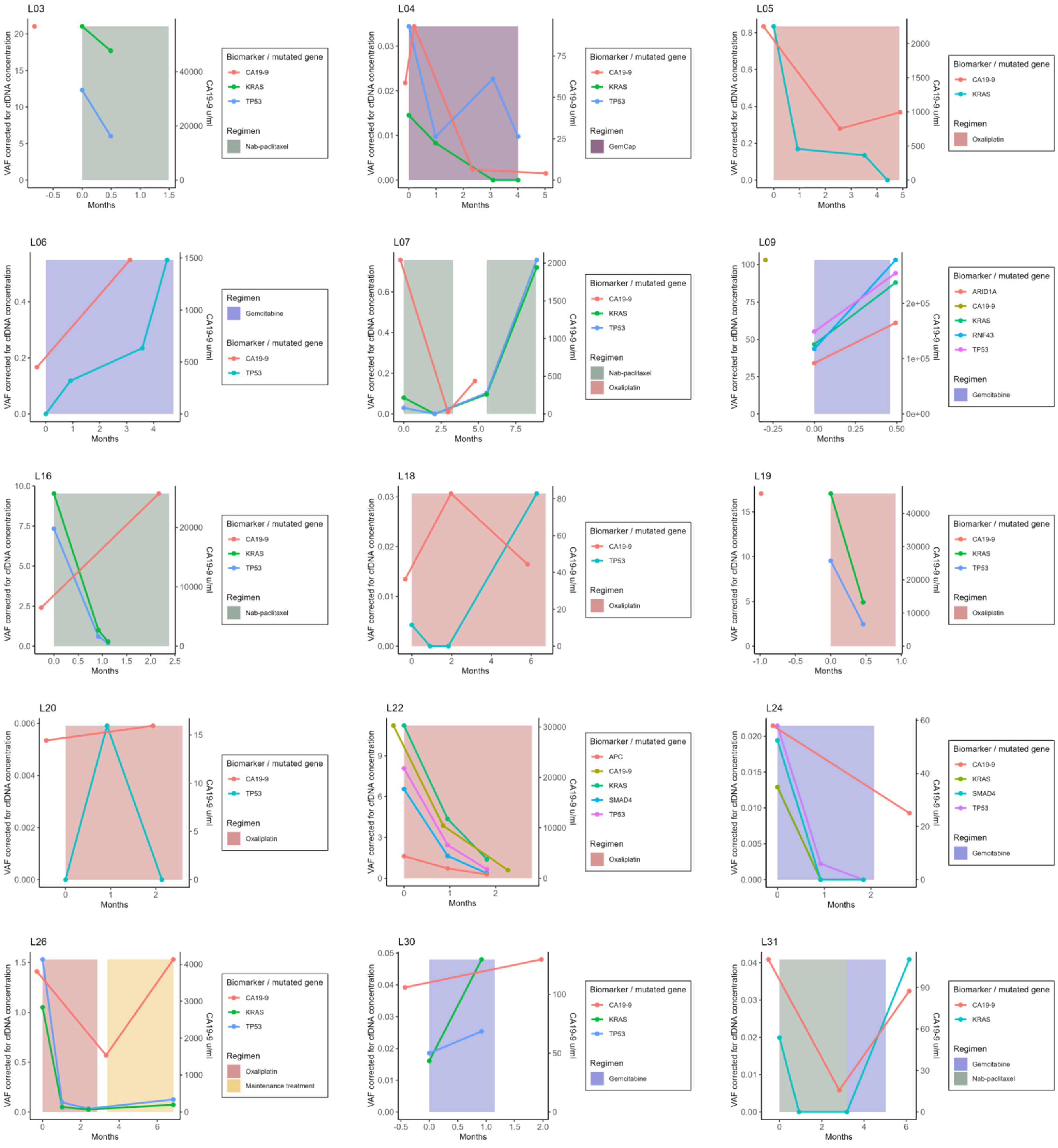

L03
L04
L05
L06
L07
L09
L16
L18
L19
L20
L22
L24
L26
L30
L31
VAF corrected for cfDNA concentration
CA19-9 u/ml
Months
Biomarker / mutated gene
Regimen
CA19-9
KRAS
TP53
APC
ARID1A
RNF43
SMAD4
Nab-paclitaxel
GemCap
Oxaliplatin
Gemcitabine
Maintenance treatment

Figure S3, continued.

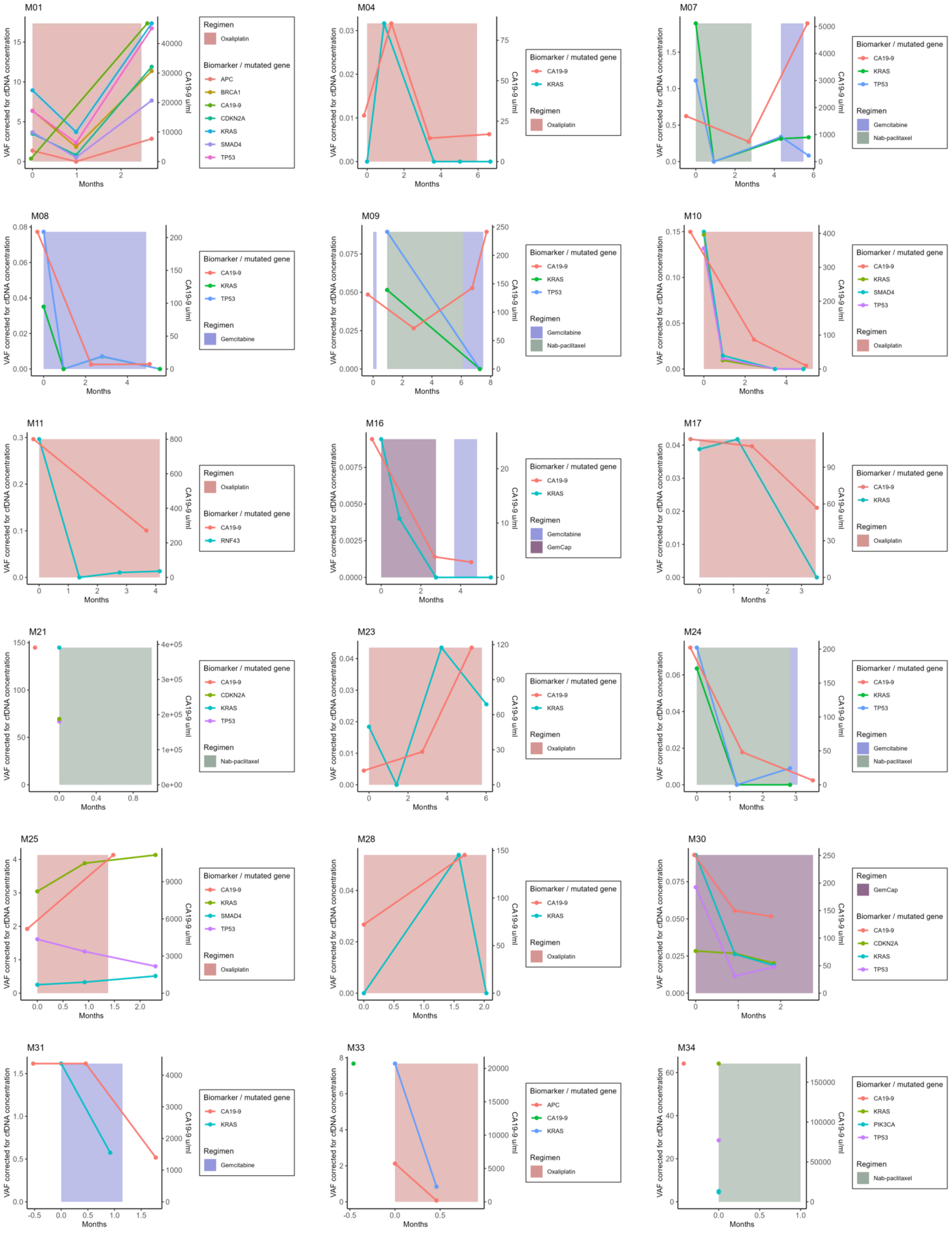

Figure S3. Trajectories for cfDNA corrected variant allele frequencies (VAF) of gene variants and levels of CA19-9 in plasma for patients with at least one ctDNA positive sample not represented in Figure 3C.

Figure S4

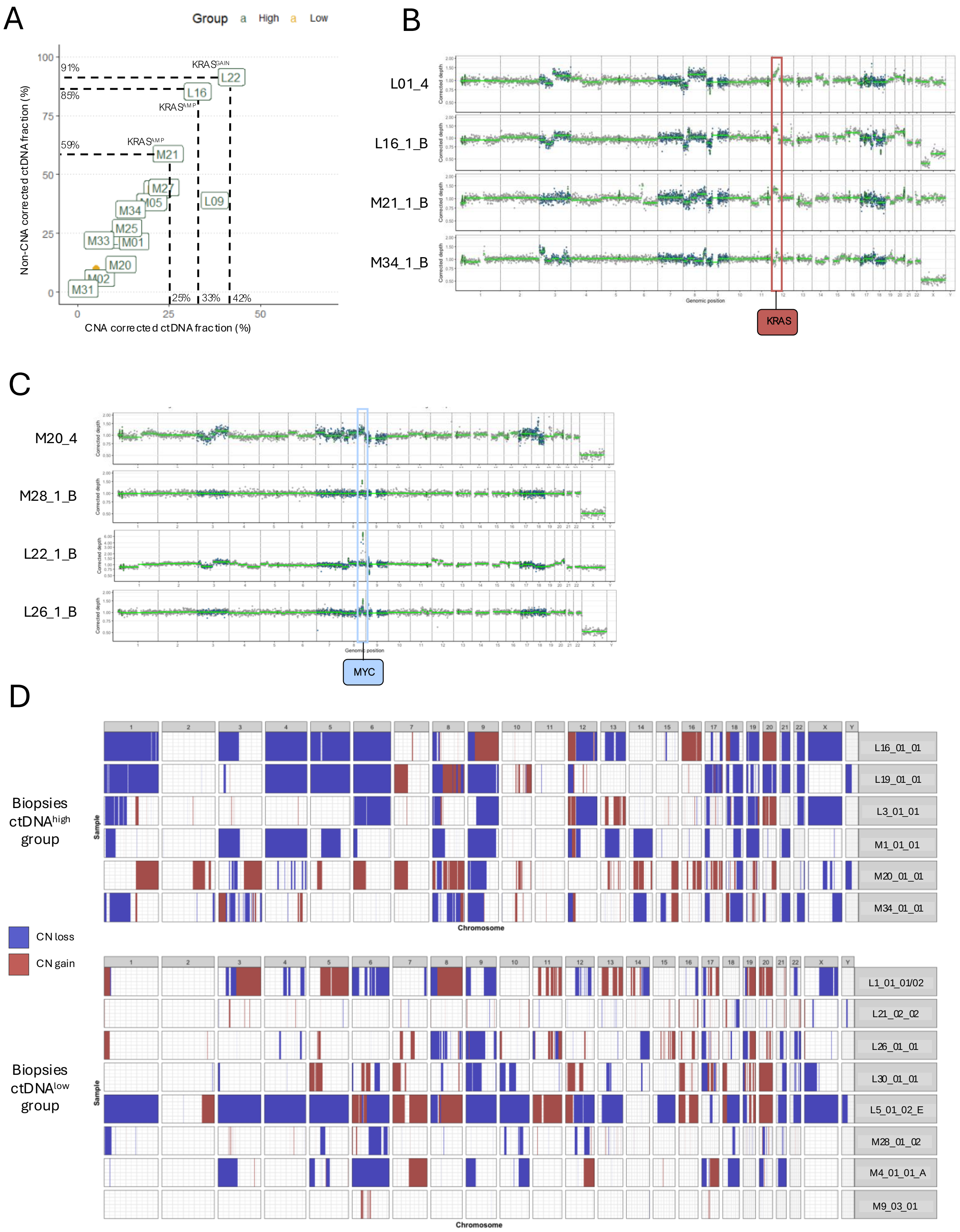

Figure S4. A) ctDNA fractions based on CNA corrected or non-corrected maximum allele frequencies. Green and yellow marks patient in the ctDNA^{high} and ctDNA^{low} groups, respectively. **B-C)** ctDNA copy number profiles highlighting amplifications of *KRAS* and *MYC*. **D)** Sample wise copy number profiles from biopsies originating from patients in the ctDNA^{high} and ctDNA^{low} group. Blue marks chromosomal losses and red chromosomal gains.

Figure S5

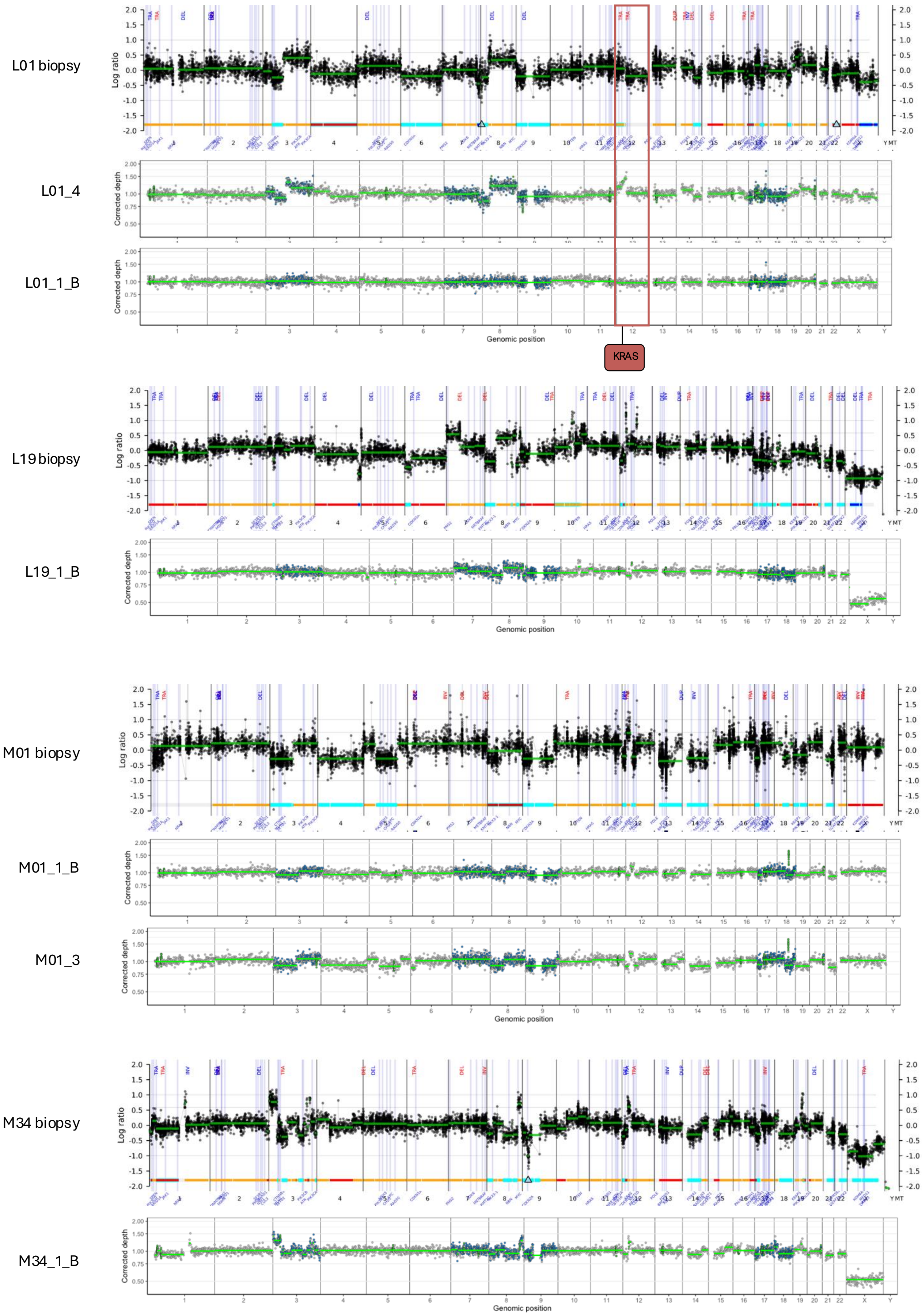

Figure S5. Comparisons of copy number profiles in matched ctDNA and tumor tissue. For patient L01, a discrepancy in regards to a *KRAS* amplification is highlighted.

Figure S6

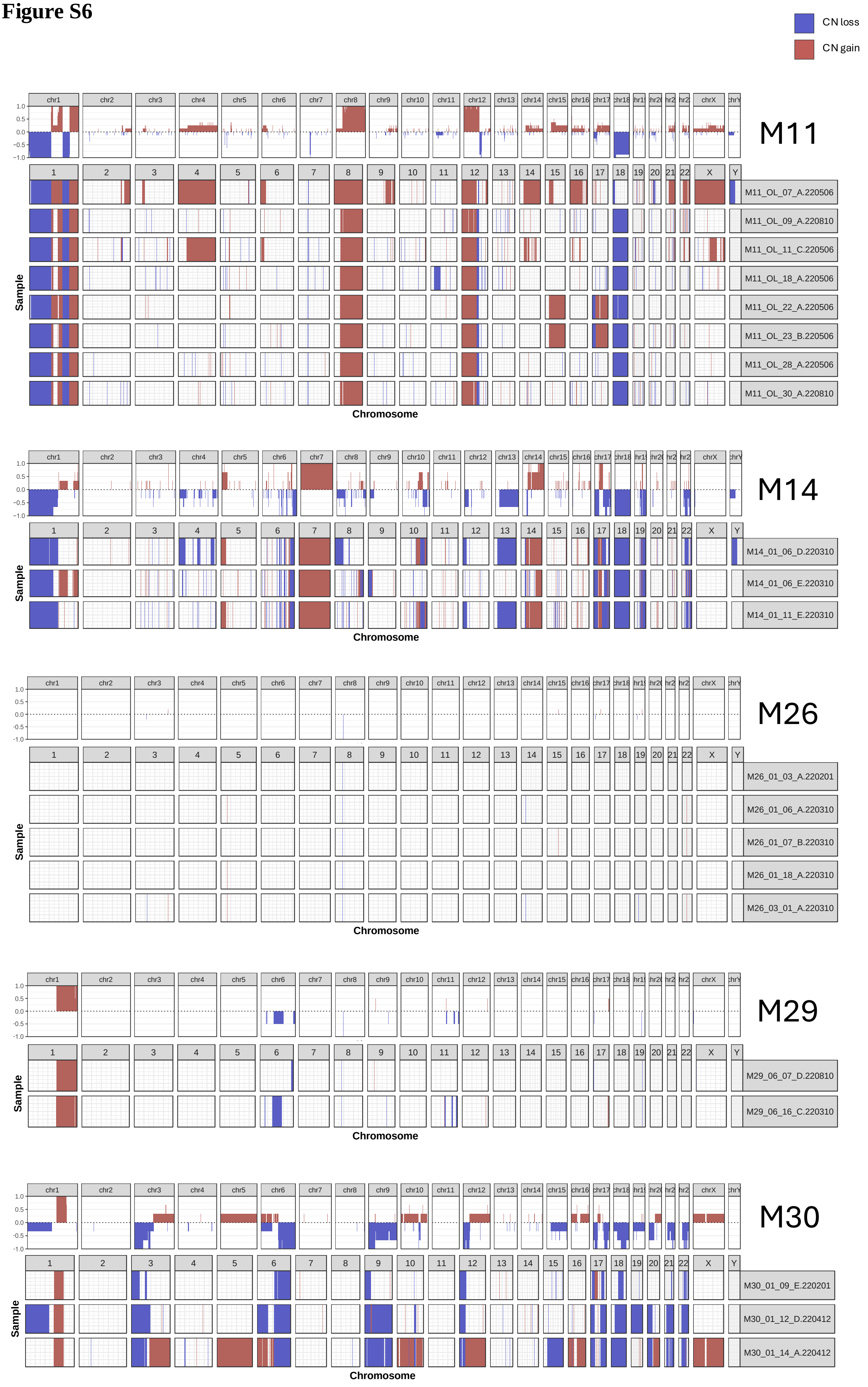

CN loss
CN gain
M11
M11_OL_07_A.220506
M11_OL_09_A.220810
M11_OL_11_C.220506
M11_OL_18_A.220506
M11_OL_22_A.220506
M11_OL_23_B.220506
M11_OL_28_A.220506
M11_OL_30_A.220810
M14
M14_01_06_D.220310
M14_01_06_E.220310
M14_01_11_E.220310
M26
M26_01_03_A.220201
M26_01_06_A.220310
M26_01_07_B.220310
M26_01_18_A.220310
M26_03_01_A.220310
M29
M29_06_07_D.220810
M29_06_16_C.220310
M30
M30_01_09_E.220201
M30_01_12_D.220412
M30_01_14_A.220412
Sample
Chromosome

Figure S6, continued.

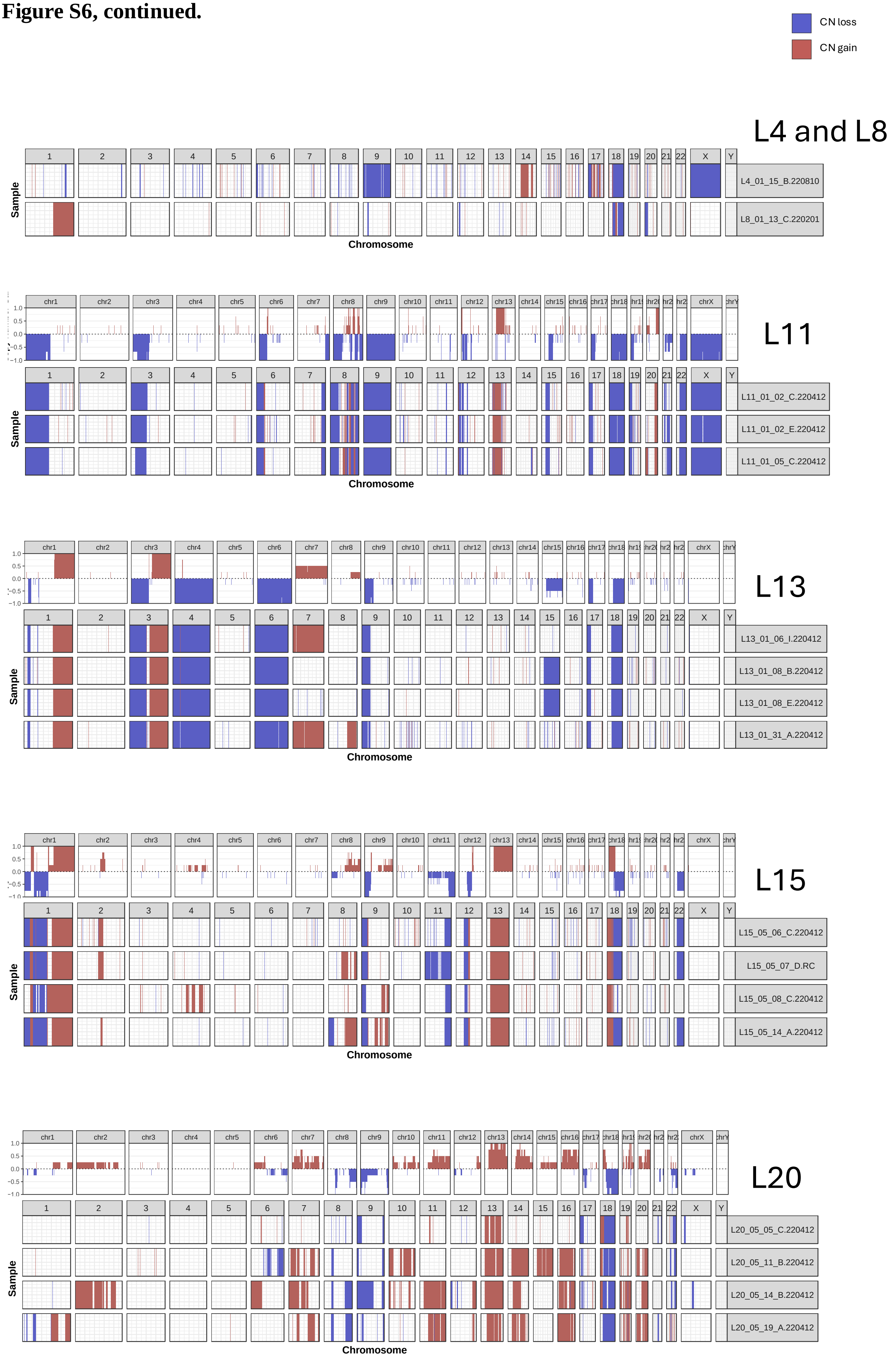

Figure S6, continued.

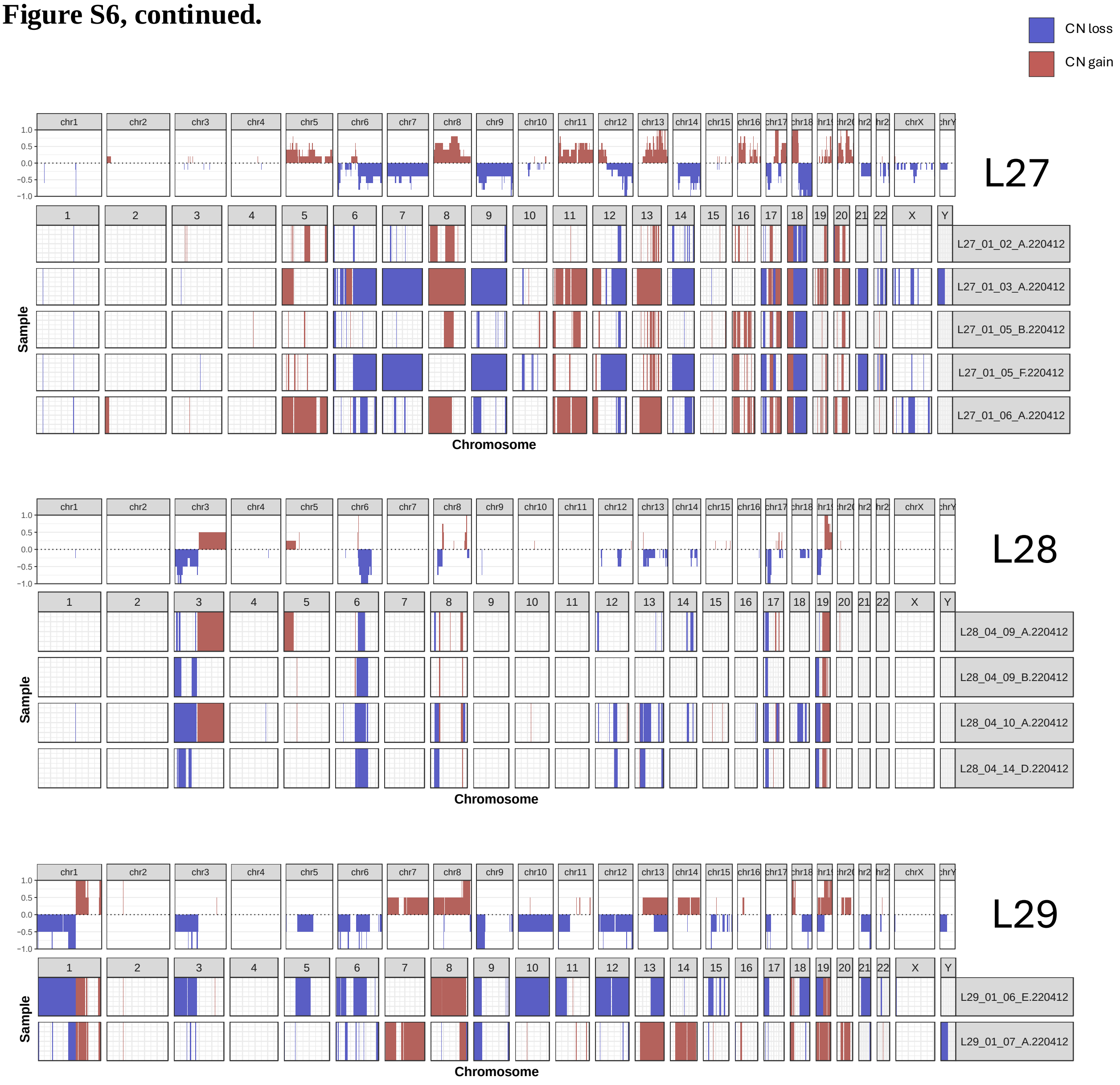

Figure S6. Multiregional copy number profiles for resected tumors and one autopsied case (M11). Blue marks chromosomal losses and red chromosomal gains.
